# Deletion of Wiskott–Aldrich syndrome protein triggers Rac2 activity and increased cross-presentation by dendritic cells

**DOI:** 10.1038/ncomms12175

**Published:** 2016-07-18

**Authors:** Marisa A. P. Baptista, Marton Keszei, Mariana Oliveira, Karen K. S. Sunahara, John Andersson, Carin I. M. Dahlberg, Austen J. Worth, Agne Liedén, I-Chun Kuo, Robert P. A. Wallin, Scott B. Snapper, Liv Eidsmo, Annika Scheynius, Mikael C. I. Karlsson, Gerben Bouma, Siobhan O. Burns, Mattias N. E. Forsell, Adrian J. Thrasher, Susanne Nylén, Lisa S. Westerberg

**Affiliations:** 1 Department of Microbiology Tumor and Cell biology, Karolinska Institutet, Stockholm 171 77, Sweden; 2 Institute for Virology and Immunobiology, University of Würzburg, 97078 Würzburg, Germany; 3 Experimental Physiopathology, Department of Sciences/Experimental Physiopatholgy, Medical School, University of São Paulo, São Paulo, Brazil; 4 Department of Medicine Solna, Translational Immunology Unit, Karolinska Institutet and Karolinska University Hospital, Stockholm 171 76, Sweden; 5 University College London Institute of Child Health, London WC1N 1EH, UK; 6 Department of Molecular Medicine and Surgery, Karolinska Institutet, Stockholm 171 76, Sweden; 7 Department of Paediatrics, Yong Loo Lin School of Medicine, National University of Singapore, Singapore 119228, Singapore; 8 Khoo Teck Puat-National University Children's Medical Institute, The National University Health System, Singapore 119228, Singapore; 9 Gastroenterology Division, Children's Hospital, Harvard Medical School, Boston MA 02115, USA; 10 Department of Medicine Solna, Dermatology and Venereology Unit, Karolinska Institutet, Stockholm 171 76, Sweden; 11 Department of Immunology, Royal Free London NHS Foundation Trust, London NW3 2QG, UK; 12 University College London Institute of Immunity and Transplantation, London WC1E 6BT, UK; 13 Department of Clinical Microbiology, Division of Immunology, Umeå University, Umeå 901 87, Sweden

## Abstract

Wiskott–Aldrich syndrome (WAS) is caused by loss-of-function mutations in the *WASp* gene. Decreased cellular responses in WASp-deficient cells have been interpreted to mean that WASp directly regulates these responses in WASp-sufficient cells. Here, we identify an exception to this concept and show that WASp-deficient dendritic cells have increased activation of Rac2 that support cross-presentation to CD8^+^ T cells. Using two different skin pathology models, WASp-deficient mice show an accumulation of dendritic cells in the skin and increased expansion of IFNγ-producing CD8^+^ T cells in the draining lymph node and spleen. Specific deletion of WASp in dendritic cells leads to marked expansion of CD8^+^ T cells at the expense of CD4^+^ T cells. WASp-deficient dendritic cells induce increased cross-presentation to CD8^+^ T cells by activating Rac2 that maintains a near neutral pH of phagosomes. Our data reveals an intricate balance between activation of WASp and Rac2 signalling pathways in dendritic cells.

## Abstract (web summary)

Wiskott–Aldrich syndrome (WAS) is a severe X-linked primary immunodeficiency syndrome. Here, the authors show that loss of WAS gene in dendritic cells results in increased activity of Rac2, increase of phagosomal pH, and more efficient cross-presentation.

## Graphical abstract

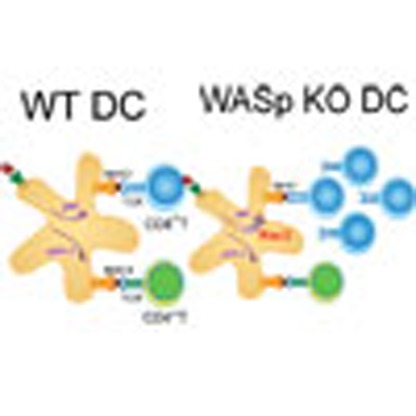

Wiskott–Aldrich syndrome (WAS) is a severe X-linked primary immunodeficiency caused by loss-of-function mutations in the gene encoding the WAS protein (WASp)^1^,^2^,^3^. More than 80% of WAS patients develop skin rash characterized as atopic eczema during infancy and childhood^1^,^2^,^3^,^4^. One possible reason for development of skin rash is the reduced function of WASp-deficient regulatory T cells that have poor suppressive activity *in vitro* and *in vivo*^5^,^6^,^7^,^8^. Surprisingly in the context of eczema, WASp-deficient mast cells have decreased capacity to degranulate upon triggering of the FcɛR1 (ref. 9). WASp-deficient Langerhans cells have decreased emigration from the epidermis upon activation with hapten^10^, and it has been suggested that skin pathology in WAS may be caused by local accumulation of dendritic cells (DCs) in the skin^1^,^10^,^11^,^12^,^13^.

WASp belongs to the WASp family of proteins also including neuronal (N)-WASp and WASp-family verprolin-homologous protein (WAVE)/suppressor of the cyclic AMP receptor (SCAR) 1-3, and they together coordinate receptor signalling to changes in the actin cytoskeleton^1^,^2^,^3^. At rest, WASp and N-WASp resides in an auto-inhibited conformation. Upon binding of the small Rho GTPase Cdc42, the auto-inhibited conformation is released and exposes the carboxy-terminal verprolin-cofilin-acidic (VCA) domain that allows for recruitment of the Arp2/3 complex and actin polymerization^14^,^15^. The small Rho GTPases Rac1 and Rac2 regulate activation of the WAVE/Scar proteins to stimulate actin polymerization by the VCA domain^16^,^17^.

Langerhans cells and CD103^+^ DCs in the skin, and CD8^+^ DCs and to a lesser extent CD8^−^ DCs in the draining lymph nodes (dLNs) and spleen possess a unique capacity for presenting exogenous antigen on major histocompatibility (MHC) class I in a process termed cross-presentation^18^,^19^,^20^. The superior capacity of specific DCs to cross-present antigens results from that DCs in contrast to other phagocytic cells can maintain a near neutral pH in phagocytic and endocytic vesicles^20^. Rac2 is a key component for cross-presentation of soluble antigens and localize to intracellular membranes in which Rac2 regulates the NADPH complex, thereby maintaining a near neutral pH in phagosomes and endosomes^21^. Moreover, CD8^−^ DCs can take up antigen in the form of immune complex by Fc receptors and efficiently shuttle exogenous antigens efficiently into the cross-presentation pathway^22^,^23^,^24^. The role of WASp in cross-presentation has been investigated by direct targeting of antigen to the DEC205 receptor expressed on CD8^+^ DCs^13^. WASp KO CD8^+^ DCs induced shorter contact duration with wild-type CD8^+^ T cells *in vivo* leading to decreased early activation of CD8^+^ T cells^13^. In the specific anti-viral response, WASp KO mice have decreased capacity to mount an antigen-specific CD8^+^ T cell response to lymphocytic choriomeningitis virus (LCMV) infection^25^ and influenza^26^,^27^.

Here, we examined the response of WASp KO mice to skin challenge. Our findings show that WASp KO mice can respond to allergens and parasite infiltration in the skin. However, the immune response is skewed to DC-mediated activation of CD8^+^ T cells that produce IFNγ. We provide evidence for that WASp KO CD8^−^ DCs upregulate the molecular machinery to cross-present antigens and activate CD8^+^ T cells. Our data suggests that downregulation of cross-presentation by WASp may be an active process that is essential to prevent over-activation of CD8^+^ T cells.

## Results

### Der p 2 induces skin pathology in WASp KO mice

**Figure 1:**
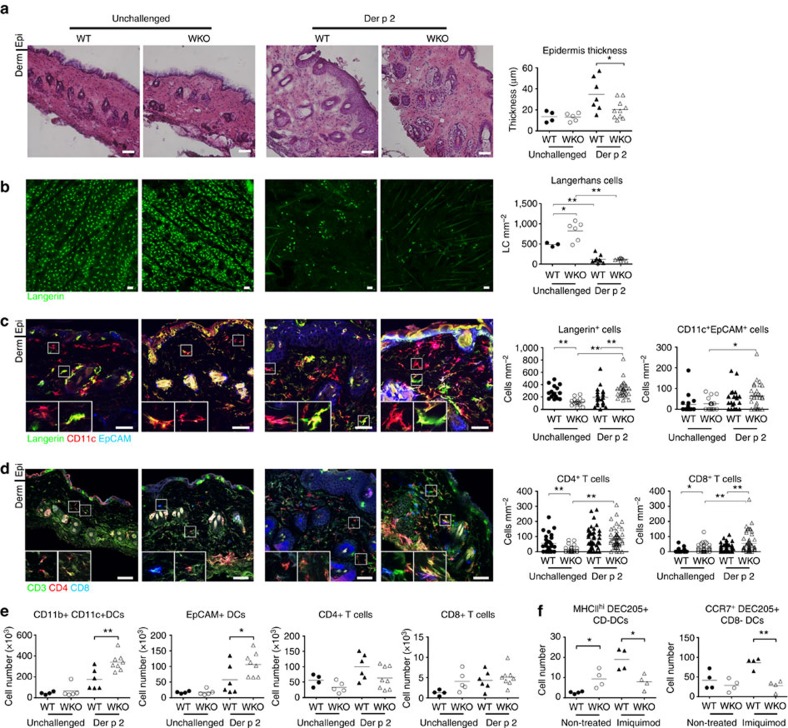
Der p 2 induces skin pathology in WASp KO mice. (**a**) Whole-skin sections (10 μm) from 4 mm^2^ punch biopsies of back skin from day 50 were stained with hematoxylin and eosin. Epidermal thickening is indicated in μm. (**b**) Langerhans cells in epidermis by histology. Epidermal sheets labelled with Langerin (CD207, green) for identification of Langerhans cells. The mean number of Langerhans cells per mm^2^ of epidermis at day 50 is indicated. (**c**,**d**) DCs and T cells in dermis by histology. (**c**) The mean number of total Langerin^+^ (including Langerhans and dermal DCs, green), CD11c^+^EpCAM^+^ (mature Langerhans cells, CD11c in red and EpCAM in blue) DCs and (**d**) CD4^+^CD3^+^ (red and green, respectively) and CD8β^+^ (blue) T cells per mm^2^ is indicated. Examples of counted cells are magnified in the white boxes. (**a**–**d**) Bar represents mean value and each dot represents one mouse (**a**,**b**) or one picture (**c**,**d**). Results are a pool of two separate experiments. (**a**–**d**) WT unchallenged *n*=3–4; WKO unchallenged *n*=3–6; WT Der p 2 *n*=3–9; WKO Der p 2 *n*=4–10. Scale bar, 50 μm. (**e**) DCs and T cells in skin by flow cytometry analysis. Absolute numbers of cells in the back skin at day 50 from unchallenged and Der p 2-challenged WT and WASp KO mice as measured by flow cytometry. WT unchallenged *n*=4; WKO unchallenged *n*=5; WT Der p 2 *n*=6; WASP KO Der p 2 *n*=8. (**f**) DC egress from the skin. Imiquimod was applied on the ear and 48 h later, MHCII^high^DEC205^+^CD8^−^ DCs and CCR7^+^DEC205^+^CD8^−^ DCs were analysed by flow cytometry in dLNs. WT unchallenged *n*=4; WKO unchallenged *n*=4; WT imiquimod *n*=4; WASP KO Imiquimod *n*=4. (**a**–**f**) Results are representative of two separate experiments. *P<0.05; **P<0.01 as calculated by the unpaired Student's *t*-test. LC, Langerhans cells; WT, wild type; WKO, WASp KO.

To induce an eczema-like phenotype, mice were shaved and treated by epicutaneous patching on the back skin with Der p 2, a major allergen from the house dust mite *Dermatophagoides pteronyssinus*^28^. Since the shaving in itself mimics mechanical injury inflicted by scratching of dry itchy skin in human eczema, we compared shaved mice patched with Der p 2 to that of unshaved unchallenged mice. To examine skin pathology at day 50, a 4 mm^2^ punch biopsy was taken from macroscopically inflamed back skin. Epidermal thickening is a hallmark of atopic dermatitis and wild-type mice showed, after three patches with Der p 2, epidermal hyperplasia (Fig. 1a)^29^,^30^ with increased number of Ki67^+^ proliferating epidermal keratinocytes (Supplementary Fig. 1a). Der p 2-challenged WASp KO mice showed less epidermal hyperplasia (Fig. 1a; Supplementary Fig. 1a). We prepared epidermal sheets and found that Langerhans cells in both wild-type mice and WASp KO mice were decreased in epidermis after Der p 2 challenge (Fig. 1b). To identify dermal DCs and Langerhans cells, we quantified the number of DC subsets in dermis including CD11c^+^EpCAM^+^ (epithelial cell adhesion molecule), Langerin^+^ and CD11c^+^EpCAM^−^Langerin^−^ DCs^18^,^31^,^32^. Wild-type mice had similar number of dermal DCs before and after Der p 2 challenge (Fig. 1c; Supplementary Fig. 1b), suggesting that the marked reduction of epidermal Langerhans cells after Der p 2 challenge in wild-type mice is caused by egress of Langerhans cells from the skin to the dLNs. Unchallenged WASp KO mice had decreased number of dermal Langerin^+^ DCs (Fig. 1c), while after Der p 2 challenge WASp KO mice had increased number of CD11c^+^EpCAM^+^, Langerin^+^ and CD11c^+^EpCAM^−^Langerin^−^ DCs in the dermis (Fig. 1c; Supplementary Fig. 1b). We detected similar number of Ki67^+^ proliferating dermal DCs in wild-type and WASp KO mice (Supplementary Fig. 1a). Wild-type mice had similar numbers of CD4^+^ and CD8^+^ T cells in the skin before and after Der p 2 challenge (Fig. 1d). WASp KO mice had decreased number of CD4^+^ T cells in unchallenged skin. Upon Der p 2 challenge, the number of CD4^+^ T cells was increased in WASp KO mice and reached similar number to Der p 2-challenged wild-type mice (Fig. 1d). In contrast, unchallenged WASp KO mice had increased number of CD8^+^ T cell in the skin compared with wild-type mice and the CD8^+^ T-cell population was further increased in WASp KO mice after Der p 2 challenge (Fig. 1d). To corroborate the results from histological analysis, we performed flow cytometry analysis of the 1 cm^2^ back skin challenged with Der p 2 (containing both macroscopically inflamed and non-inflamed skin). Upon Der p 2 challenge, wild-type and WASp KO mice had increased number of CD45^+^ hematopoietic cells in the skin when compared with unchallenged mice (Supplementary Fig. 1c). Der p 2 challenge induced increased number of CD11b^+^CD11c^+^ and EpCAM^+^ DCs in the WASp KO skin when compared with Der p 2-challenged wild-type skin (Fig. 1e). However, due to the fact that we assessed both lesional and non-lesional skin by flow cytometry, Der p 2 challenge did not induce a significant increase of CD4^+^ and CD8^+^ T cells in wild-type or WASp KO mice (Fig. 1e). WASp KO mice had fewer IFNγ-producing CD4^+^ T cells when compared with wild-type mice both before and after Der p 2 challenge (Supplementary Fig. 1c). Moreover, WASp KO mice had a tendency to increased number of IFNγ-producing CD8^+^ T cells and CD11c^+^EpCAM^−^CD103^+^ DCs (Supplementary Fig. 1c).

On treatment with the TLR7 agonist imiquimod, wild-type mice exhibited increased numbers of migratory MHCII^high^DEC205^+^CD8^−^ DCs and CCR7^+^DEC205^+^CD8^−^ DCs in the dLNs, whereas WASp KO DCs failed to migrate to the dLNs (Fig. 1f). This implied that WASp KO skin DCs had decreased capacity to egress and therefore accumulated in the dermis.

**Figure 2:**
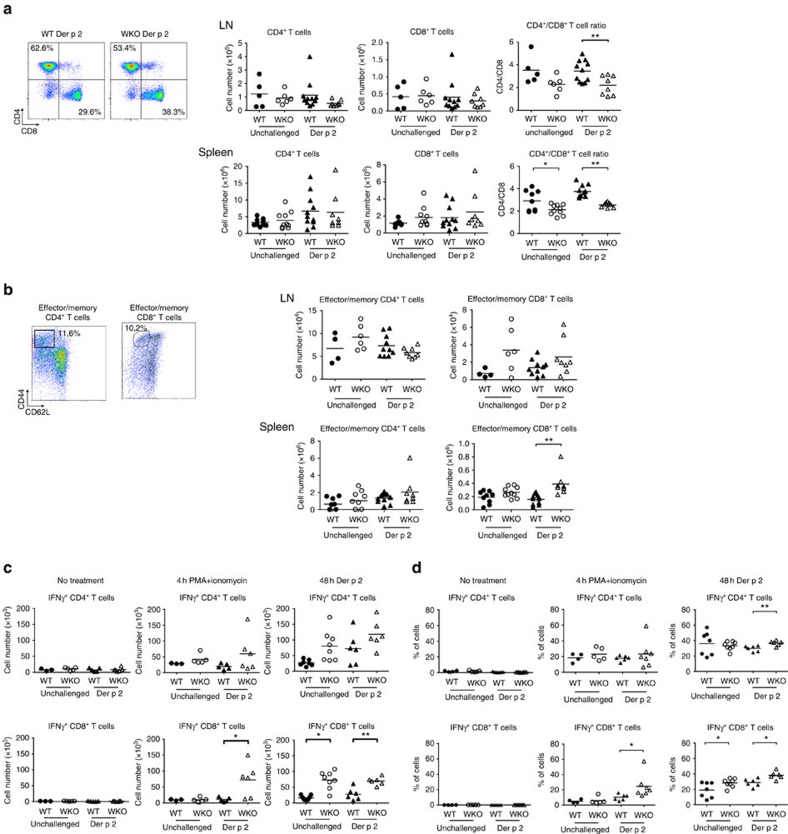
Der p 2 induces expansion of WASp KO CD8^+^IFNγ^+^ T cells. (**a**,**b**) LN and spleen T cells by flow cytometry. Absolute numbers of (**a**) total and (**b**) effector/memory (CD44^hi^CD62L^−^), CD4^+^ and CD8^+^ T cells from day 50 spleens and dLNs from unchallenged and Der p 2-challenged WT and WASp KO mice on Balb/c background as measured by flow cytometry. (**c**) *In vitro* stimulation of spleen cells. Total splenocytes from unchallenged or Der p 2-challenged mice at day 50 were either unstimulated or stimulated with PMA plus ionomycin for 4 h or Der p 2 for 48 h (**c**). Absolute numbers of total CD4^+^IFNγ^+^ and CD8^+^IFNγ^+^ T cells after Der p 2 and PMA plus ionomycin stimulation as measured by flow cytometry. (**a**–**c**) Bar represents mean value and each dot represents one mouse. (**a**,**b**) Results are a pool of two separate experiments and (**c**) representative of two separate experiments. (**a**,**b**) WT unchallenged *n*=4–9; WKO unchallenged *n*=6–10; WT Der p 2 *n*=10–11; WKO Der p 2 *n*=8. (**c**) WT unchallenged *n*=3–7; WKO unchallenged *n*=5–8; WT Der p 2 *n*=5–6; WKO Der p 2 *n*=6. *P<0.05; **P<0.01 as calculated by the unpaired Student's *t*-test. WT, wild type; WKO, WASp KO.

When we analyzed the Der p 2 response in dLNs and spleen, individual WASp KO mice showed a consistent increase in CD8^+^ T cells over CD4^+^ T cells leading to a skewed CD4/CD8 T-cell ratio, although the mean total number of LN and spleen CD4^+^ and CD8^+^ T cells were similar in wild-type and WASp KO mice (Fig. 2a). Compared to LNs and spleen, WASp KO mice had a reversed CD4/CD8 ratio in blood, suggesting that WASp KO CD8^+^ T cells preferentially accumulated in tissues (Supplementary Fig. 2a). Wild-type and WASp KO mice had similar number of memory/effector CD4^+^ T cells before and after Der p 2 challenge as determined by CD44^high^/CD62L^−^ cells (Fig. 2b), but Der p 2 challenge induced higher number of memory/effector CD8^+^ T cells in the spleen of WASp KO mice when compared with wild-type mice (Fig. 2b).

Together, these results suggests that Der p 2-challenged WASp KO mice had an accumulation of DCs in the dermis and an altered systemic T-cell balance with increased number of effector/memory CD8^+^ T cells.

### Der p 2 induces expansion of WASp KO CD8^+^IFNγ^+^ T cells

To examine the cytokine profile in WASp KO mice, we measured cytokines in serum and skin. Similar quantities of TNFα, IL-4, IL-5, IL-6, IL-10, IL-13, IFNγ and TGFβ in wild-type and WASp KO mice were detected both before and after Der p 2 challenge (Supplementary Fig. 2b). When we examined the CD8^+^ T cells, there was no detectable IFNγ production in freshly isolated cells from either wild-type or WASP KO mice (Fig. 2c,d). However, after 4 h stimulation with phorbol 12-myristate 13-acetate (PMA) and ionomycin, Der p 2-challenged WASp KO mice exhibited a high proportion of IFNγ-producing CD8^+^ T cells (Fig. 2c,d). In splenocytes stimulated for 48 h with Der p 2 to examine the allergen-specific response, both unchallenged and Der p 2-challenged WASp KO mice had increased number of IFNγ-producing CD8^+^ T cells (Fig. 2c,d). WASp KO T cells from unchallenged mice, not previously stimulated with Der p 2, also showed expansion of CD8^+^IFNγ^+^ T cells upon Der p 2 treatment *in vitro.* Since few naive T cells will contain the Der p 2 specificity, this suggests that naive WASp KO CD8^+^ T cells, but not CD4^+^ T cells, were prone to produce IFNγ irrespective of antigen specificity.

### Increased WASp KO CD8^+^IFNg^+^ T cells upon *L. major* infection

**Figure 3:**
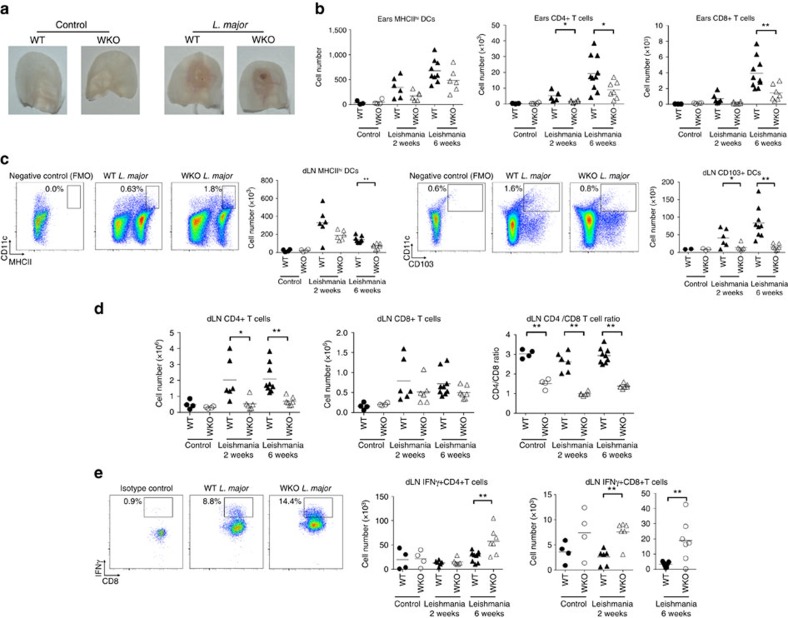
*L. major* induces increased number of WASp KO CD8^+^IFNγ^+^ T cells. (**a**) Ear infiltration of cells. (**a**) Ears from WT and WASp KO control or *L. major* infected mice on Balb/c background after 6 weeks. (**b**) Absolute numbers in ear of total MHCII^hi^CD11c^+^ DCs; total CD4^+^CD3^+^ and CD8^+^CD3^+^ T cells, measured by flow cytometry. (**c**–**e**) dLN infiltration of cells. Absolute numbers in dLN of total MHCII^hi^ DCs; total CD4^+^CD3^+^ and CD8^+^CD3^+^ T cells; CD4^+^/CD8^+^ T-cell ratio; IFNγ^+^CD4^+^CD3^+^ and IFNγ^+^CD8^+^CD3^+^ cells, measured by flow cytometry. (**a**–**e**) Bar represents mean value and each dot represents one ear or dLNs. Results from week 2 and week 6 are representative of two separate experiments. WT control *n*=3–4; WKO control *n*=4; WT *L. major* 2 weeks *n*=6; WASp KO *L. major* 2 weeks *n*=6; WT *L. major* 6 weeks *n*=10; WASp KO *L. major* 6 weeks *n*=7. *P<0.05; **P<0.01 as calculated by the unpaired Student's *t*-test. FMO, fluorescence minus one (negative control for MHC class II and CD103); WT, wild type; WKO, WASp KO.

We next investigated how WASp KO mice would respond to dermal infection. *Leishmania major (L. major)* infect dermal macrophages and induce a massive Th1 response characterized by CD4^+^ T cells producing IFNγ^33^,^34^. When compared with wild-type mice, WASp KO mice had a delayed response to *L. major* infection at 2 weeks post infection as evidenced by smaller lesion size (Fig. 3a; Supplementary Fig. 3a) and decreased CD4^+^ T-cell infiltration (Fig. 3b). At 6 weeks post *L. major* infection, both wild-type and WASp KO mice had large lesions (Fig. 3a; Supplementary Fig. 3a) with considerable infiltration of MHC class II^hi^ DCs, CD4^+^ and CD8^+^ T cells and macrophages (Fig. 3b; Supplementary Fig. 3b,c). At 6 weeks, dLNs in wild-type mice had increased number of MHC class II^high^ DCs, which had likely emigrated from the infected skin (Fig. 3c). Moreover, wild-type mice had increased numbers of CD103^+^, CD8α^+^ and CD8α^−^ DCs capable of cross-presenting exogenous antigen and activate CD8^+^ T cells (Fig. 3c; Supplementary Fig. 3d). In contrast, WASp KO mice showed no increased numbers of MHC class II^high^ DCs or CD103^+^, CD8α^+^ and CD8α^−^ DCs in the dLNs upon infection (Fig. 3c; Supplementary Fig. 3d). Together with increased accumulation of DCs in the dermis of WASp KO mice after Der p 2 challenge, this suggests that WASp KO DCs have decreased capacity to egress from dermis.

In the T-cell compartment of dLNs, WASp KO mice had significantly lower number of CD4^+^ T cells both at 2 and 6 weeks post infection when compared with wild-type mice (Fig. 3d). While the total number of CD8^+^ T cells was similar in wild-type and WASp KO dLNs upon *L. major* infection, WASp KO mice showed a consistent failure to accumulate CD4^+^ T cells in dLNs leading to a skewed CD4/CD8 T-cell ratio irrespective of *L. major* infection (Fig. 3d). We detected similar number of IFNγ-producing CD4^+^ and CD8^+^ T cells in the dLNs of wild-type mice before and after *L. major* infection (Fig. 3e). In contrast, WASp KO mice had increased number of IFNγ-producing CD4^+^ and CD8^+^ T cells in the dLNs (Fig. 3e). Together, this data suggests that WASp KO mice, despite having less antigen-presenting DCs in dLNs, can activate IFNγ-producing CD4^+^ and CD8^+^ T cells upon *L. major* infection.

### DC-specific WASp deletion induces increased CD8^+^ T cells

**Figure 4:**
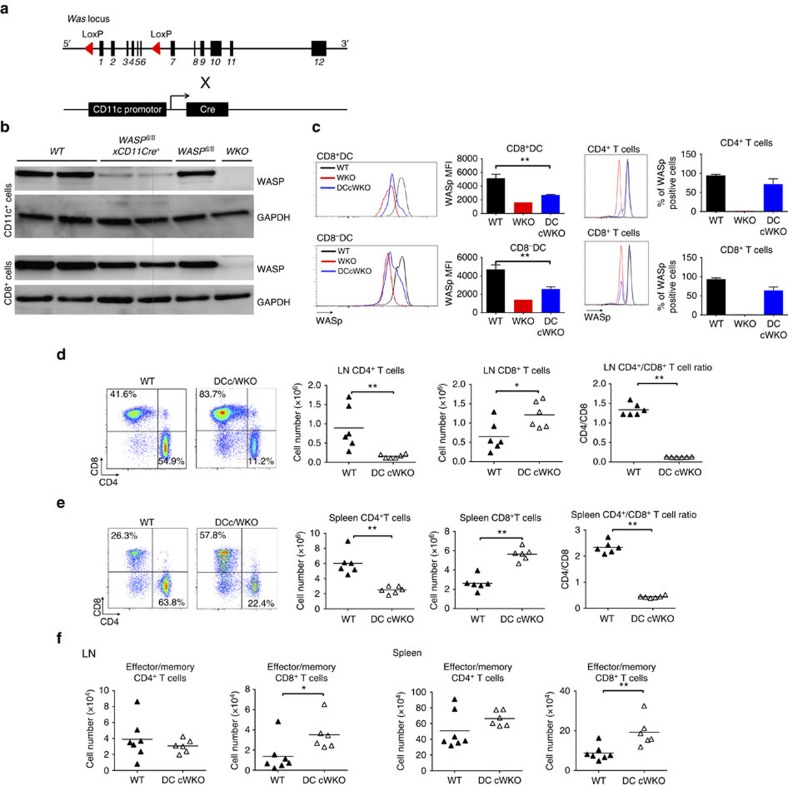
DC-specific WASp deletion induces increased CD8^+^ T cells. (**a**) Schematic representation of the targeting strategy. Mice containing the *WAS* allele flanked by *loxP* sites were bred with CD11c-Cre mice to generate DC/cWKO mice on C57Bl/6 background. (**b**,**c**) WASp expression as determined by (**b**) western blotting and (**c**) flow cytometry of CD11c^+^CD8^+^, CD11c^+^CD8^−^, CD4^+^CD3^+^ and CD8^+^CD3^+^ cells from spleen. (**d**,**e**) Flow cytometry analysis of total CD4^+^CD3^+^ and CD8^+^CD3^+^ T cells in the (**d**) LNs and (**e**) spleen and (**f**) total effector/memory (CD44^hi^CD62L^−^) CD4^+^CD3^+^ and CD8^+^CD3^+^ T cells in LNs and spleen. **(c**) Bar represents mean±s.d. of WT *n*=3; WKO *n*=1; DC/cWKO *n*=3. (**d**,**f**) WT *n*=6–7; DC/cWKO *n*=6. The data is representative of (**b**) one, (**c**) two, (**d**,**e**) four and (**f**) two separate experiments. *P<0.05; **P<0.01 as calculated by the unpaired Student's *t*-test. DC/cWKO, WASp^fl/fl^CD11c^Cre/wt^; fl, floxed (LoxP flanked); WT, wild type; WKO, WASp KO.

To determine if WASp KO DCs induce expansion of wild-type CD8^+^ T cells, we took advantage of mice harbouring a conditionally targeted *loxP*-flanked WASp allele^35^ bred to mice expressing Cre recombinase under the DC-expressed CD11c promoter (CD11c^Cre/wt^ mice), referred to as DC/cWKO mice (Fig. 4a). In DC/cWKO mice, WASp expression was efficiently deleted in CD11c^+^ DCs and a large fraction of CD8^+^ and CD8^−^ DCs had decreased to absent expression of WASp (Fig. 4b,c; images in Fig. 4b have been cropped for presentation. Full size images are presented in Supplementary Fig. 4). CD11c-driven deletion of WASp led to the absence of WASp in 10% of CD4^+^ T cells and 30% of CD8^+^ T cells (Fig. 4b,c). When comparing maturation and activation phenotype to wild-type CD8^+^ DCs, DC/cWKO CD8^+^ DCs had lower expression of MHC class II and CD86, while expression of MHC class I and II, and CD86 was similar in wild-type and DC/cWKO CD8^−^ DCs (Supplementary Fig. 5). When compared with wild-type mice, DC/cWKO mice had increased numbers of CD8^+^ T cells and decreased number of CD4^+^ T cells both in the LNs and spleen, creating a skewed CD4/CD8 T-cell ratio (Fig. 4d,e). Moreover, DC/cWKO mice had increased number of effector/memory CD8^+^ T cells both in the LNs and spleen when compared with wild-type mice while effector/memory CD4^+^ T cells were similar in wild-type and DC/cWKO mice (Fig. 4f). This data indicates that reduced expression of WASp in CD11c^+^ DCs was associated with expansion and activation of wild-type CD8^+^ T cells.

### Increased cross-presentation by WASp KO DCs

**Figure 5:**
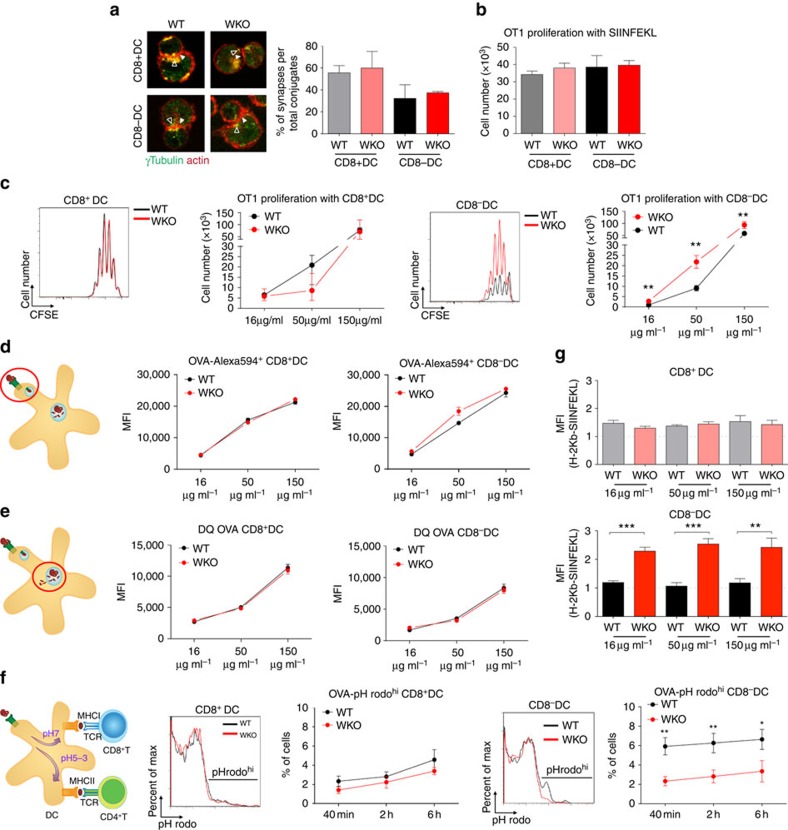
Increased cross-presentation by WASp KO DCs. (**a**) Immune synapse. Enriched CD8^+^ and CD8^−^ DCs from Flt3L tumour cell-injected mice on C57Bl/6 background were pulsed with ovalbumin and incubated with OT-I CD8^+^ T cells. Percentage of synapses was measured by counting the number of conjugates with polarized actin (red) and microtubule organizing center γ-tubulin (green) towards the synapse and divided by the total number of cell conjugates. (**b**) CD8^+^ T-cell proliferation with SIINFEKL peptide. Enriched CD8^+^ and CD8^−^ DCs from Flt3L tumour cell-injected mice on C57Bl/6 background were incubated overnight with 2 μg ml^−1^ SIINFEKL peptide and co-cultured with CFSE-labelled OT-I (Vβ5.1/5.2^+^) CD8^+^ T cells for 72 h. Total number of OT-I CD8^+^ T cells is indicated. (**c**) CD8^+^ T-cell proliferation with ovalbumin. Equal numbers of FACS-sorted splenic CD8^+^ DCs and CD8^−^ DCs from wild-type and WASp KO mice on C57Bl/6 background were incubated overnight with ovalbuminm, co-cultured with CFSE-labelled OT-I (Vβ5.1/5.2^+^) CD8^+^ T cells, and proliferation determined at 72 h. Total number of OT-I CD8^+^ T cells is indicated. (**d**) Ovalbumin uptake. DCs were incubated with soluble ovalbumin-Alexa594 to assess uptake of ovalbumin. (**e**) Ovalbumin degradation. DCs were incubated with soluble DQ-ovalbumin to assess the capacity to process antigen. Note that increased DQ-ovalbumin mean fluorescence intensity indicates increased degradation. (**f**) Ovalbumin acidification. DCs were incubated with soluble pH rodo-ovalbumin. Note that increased pH rodo-ovalbumin mean fluorescence intensity indicates decreased pH value. (**g**) CD8^+^ DCs and CD8^−^ DCs from wild-type and WASp KO mice were incubated overnight with ovalbumin. The presentation of SIINFEKL peptide on MHC class I H-2K^b^ molecules was assessed by flow cytometry and fold increase in expression was determined using the MFI value acquired for 0 μg ml^−1^ ovalbumin set to 1 (dotted line) (**a**) A total of 39–113 conjugates per mouse was analyzed. (**a**–**g**) Bar represents mean±s.d. of WT *n*=3; WKO *n*=3 per group. The data is representative of (**a**,**b**,**g**) two experiments, (**c**) four experiments and (**d**–**f**) three separate experiments. *P<0.05; **P<0.01 as calculated by the unpaired Student's *t*-test. MFI, mean fluorescence intensity; WT, wild type; WKO, WASp KO.

The increased expansion of CD8^+^ T cells in WASp KO and DC/cWKO mice led us to investigate if WASp KO DCs would have increased capacity to cross-present exogenous antigen and activate CD8^+^ T cells. To address this possibility, wild-type CD8^+^ T cells from OT-I TCR transgenic mice were used in which all CD8^+^ T cells recognize the SIINFEKL peptide from ovalbumin presented by H-2K^b^ MHC class I molecules. Wild-type and WASp KO CD8^+^ DCs and CD8^−^ DCs, loaded with soluble ovalbumin, had similar capacity to form immune synapses with OT-I CD8^+^ T cells (Fig. 5a). When comparing direct presentation of the SIINFEKL peptide, we detected similar proliferation of OT-I CD8^+^ T cells when stimulated with wild-type or WASp KO CD8^+^ or CD8^−^ DCs (Fig. 5b). These findings suggest that WASp KO DCs could form immune synapses with CD8^+^ T cells to facilitate their activation. To examine the cross-presenting capacity, we compared CD8^+^ DCs with high capacity to cross-present soluble antigens and CD8^−^ DCs with less capacity to cross-present soluble antigens^21^. Wild-type and WASp KO CD8^+^ DCs had similar capacity to induce OT-I CD8^+^ T-cell proliferation in response to soluble ovalbumin (Fig. 5c). In contrast, WASp KO CD8^−^ DCs induced increased OT-I CD8^+^ T-cell proliferation when compared with wild-type CD8^−^ DCs as determined by dilution of CFSE (Fig. 5c) and ^3^H-thymidine incorporation (Supplementary Fig. 5). CD8^−^ DCs, and to a lesser extent CD8^+^ DCs, can efficiently take up antigen in the form of immune complex by Fc receptors and shuttle the antigen into the cross-presentation pathway^23^,^24^. When compared with wild-type CD8^−^ DCs, WASp KO CD8^−^ DCs had reduced capacity to cross-present antigen from immune complexes leading to decreased proliferation of OT-I CD8^+^ T cells (Supplementary Fig. 7a–c). We next examined antigen processing and presentation on MHC class II using soluble ovalbumin. Wild-type and WASp KO CD8^+^ had similar capacity to stimulate proliferation of ovalbumin-specific OT-II CD4^+^ T cells. In contrast, WASp KO CD8^−^ DCs induced less proliferation when compared with wild-type CD8^−^ DCs (Supplementary Fig. 8). This suggests that WASp KO CD8^−^ DCs favoured presentation of soluble ovalbumin on MHC class I molecules.

We next tried to identify the cause for the increased capacity of WASp KO CD8^−^ DCs to cross-present soluble ovalbumin and stimulate proliferation of CD8^+^ T cells. Wild-type and WASp KO DCs had similar maturation phenotype as assessed by upregulation of CD86 and MHC class I molecules (Supplementary Fig. 5); normal uptake of soluble ovalbumin (Fig. 5d); and normal degradation of ovalbumin as determined by usage of DQ-OVA that start to emit fluorescence when ovalbumin is processed in the cell (Fig. 5e). For cross-presentation to occur, DCs need to maintain a near neutral pH of the phagosome that allows for escape of proteins to the cytosol and loading on MHC class I molecules^20^. To examine acidification, we used pH rodo-ovalbumin that emits fluorescence at low pH (pH 3–5), indicating when ovalbumin reaches lysosomes. Wild-type CD8^+^ DCs had a lower percentage of pHrodo^high^ cells when compared with wild-type CD8^−^ DCs (Fig. 5f), showing the low capacity of CD8^+^ DCs to acidify endosomes. WASp KO CD8^+^ DCs showed similar low percentage of pHrodo^high^ cells as wild-type CD8^+^ DCs (Fig. 5f). In contrast to wild-type CD8^−^ DCs with increased percentage of pHrodo^high^ cells, WASp KO CD8^−^ DCs had decreased percentage of pHrodo^high^ cells (Fig. 5f). To evaluate presentation of ovalbumin peptides on MHC class I molecules, DCs were incubated with soluble ovalbumin and labelled with an antibody specific for the ovalbumin peptide SIINFEKL presented by MHC class I molecule H-2K^b^. Wild-type and WASp KO CD8^+^ DCs had similar expression of SIINFEKL–H-2K^b^ on the surface (Fig. 5g). When compared with wild-type CD8^−^ DCs, WASp KO CD8^−^ DCs presented more SIINFEKL in the context of H-2K^b^ (Fig. 5g).

**Figure 6:**
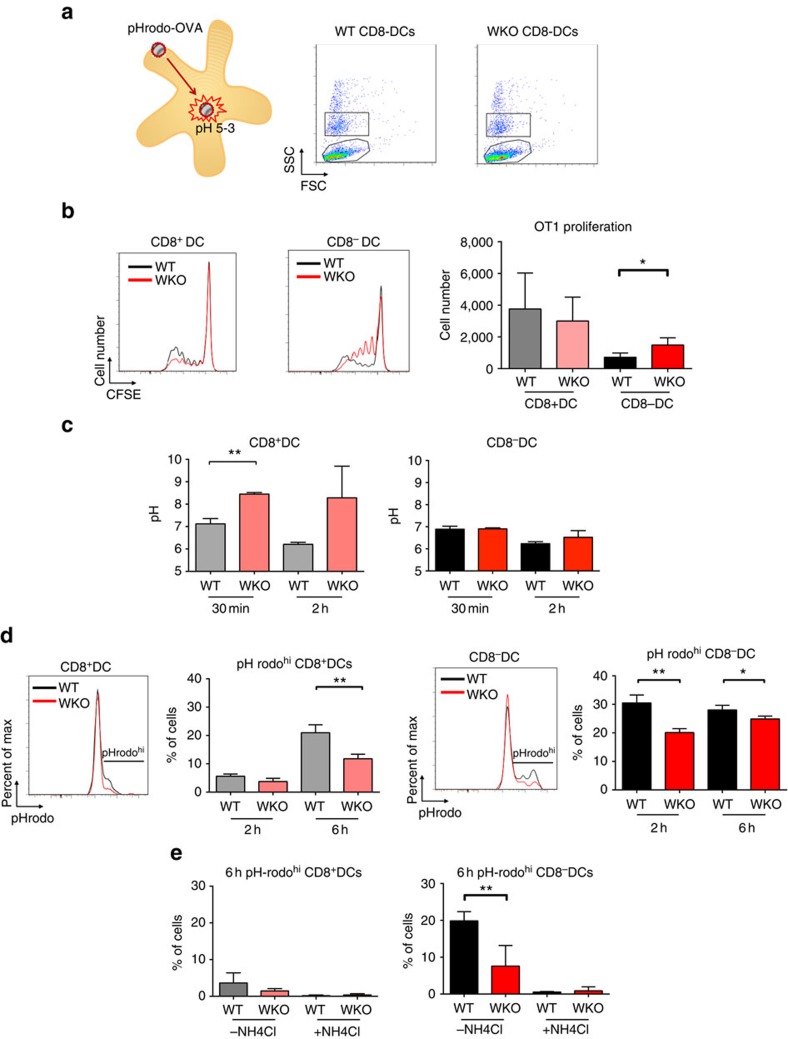
The phagosomal compartment in WASp KO DCs. Wild-type and WASp KO CD8^+^ and CD8^−^ DCs from mice on C57Bl/6 background were incubated with ovalbumin-coated latex beads overnight. (**a**) Ovalbumin-bead uptake by DCs. (**b**) CD8^+^ T-cell proliferation with ovalbumin-coated beads. FACS-sorted DCs that had taken up one ovalbumin-coated bead were co-cultured with CFSE-labelled OT-I (Vβ5.1/5.2^+^) CD8^+^ T cells, and proliferation determined as CFSE dilution at 72 h. (**c**) DCs were incubated with latex beads coupled with pH-sensitive (FITC) and pH-insensitive (Alexa647) dyes. FITC and Alexa647 intensities were measured at the specified time points and the pH was determined as described in the materials and methods. (**d**,**e**) Ovalbumin-bead acidification. (**d**) DCs were incubated with pH rodo-ovalbumin-coated beads to assess acidification of antigen in phagocytic vesicles. Note that increased pH rodo mean fluorescence intensity translates into decreased pH value. (**e**) DCs were pre-treated with NH_4_Cl to abolish acidification before addition of pH rodo-ovalbumin beads. (**b**–**e**) Bar represents mean±s.d. of WT *n*=3; WKO *n*=3. The data are representative of (**b**,**d**) three and (**c**,**e**) two separate experiments. *P<0.05; **P<0.01 as calculated by the unpaired Student's *t*-test. WT, wild type; WKO, WASp KO.

To precisely determine the cross-presentation and acidification capacity of DCs, we used latex beads that were coated with ovalbumin (Fig. 6a). To circumvent differences in uptake of beads, DCs that had taken up one ovalbumin-coated bead (Fig. 6a) were sorted by flow cytometry and examined for their capacity to induce cross-presentation. Wild-type and WASp KO CD8^+^ DCs induced high proliferation of OT-I CD8^+^ T cells when compared with wild-type CD8^−^ DCs (Fig. 6b). Again, WASp KO CD8^−^ DCs induced higher proliferation of OT-I CD8^+^ T cells when compared with wild-type CD8^−^ DCs (Fig. 6b). To assess if increased cross-presentation by WASp KO CD8^−^ DCs was associated with changes in acidification upon phagocytosis, we first examined the early acidification events using beads bearing a mixture of pH-sensitive (FITC) and pH-insensitive (Alexa-fluor647) dyes that allows detection of phagosomal pH 5–8 (ref. 36). Using this approach, WASp KO CD8^+^ DCs maintained their phagosomes at a higher pH during the first 2 h after phagocytosis when compared with wild-type CD8^+^ DCs (Fig. 6c). For CD8^−^ DCs, wild-type and WASp KO DCs showed similar phagosomal pH at 30 min and 2 h (Fig. 6c). To examine the later acidification events, we used beads coated with pH rodo-ovalbumin that measures phagolysosomal pH 3–5. When compared with wild-type CD8^+^ DCs, WASp KO CD8^+^ DCs showed decreased percentage of pHrodo^high^ cells after 6 h (Fig. 6d). Also for CD8^−^ DCs, WASp KO CD8^−^ DCs showed lower percentage of pHrodo^high^ cells when compared with wild-type CD8^−^ DCs (Fig. 6d). To confirm that the pH rodo fluorescence represented acidification, cells were pre-treated with NH_4_Cl that completely abolished acidification of pH rodo-ovalbumin-coated beads (Fig. 6e). Together, this data suggests that WASp KO CD8^−^ DCs had decreased capacity to acidify endocytic and phagocytic vesicles and this was associated with cross-presentation on MHC class I and increased proliferation of CD8^+^ T cells.

### WASp KO CD8^−^ DCs activate Rac2 and ROS production

**Figure 7:**
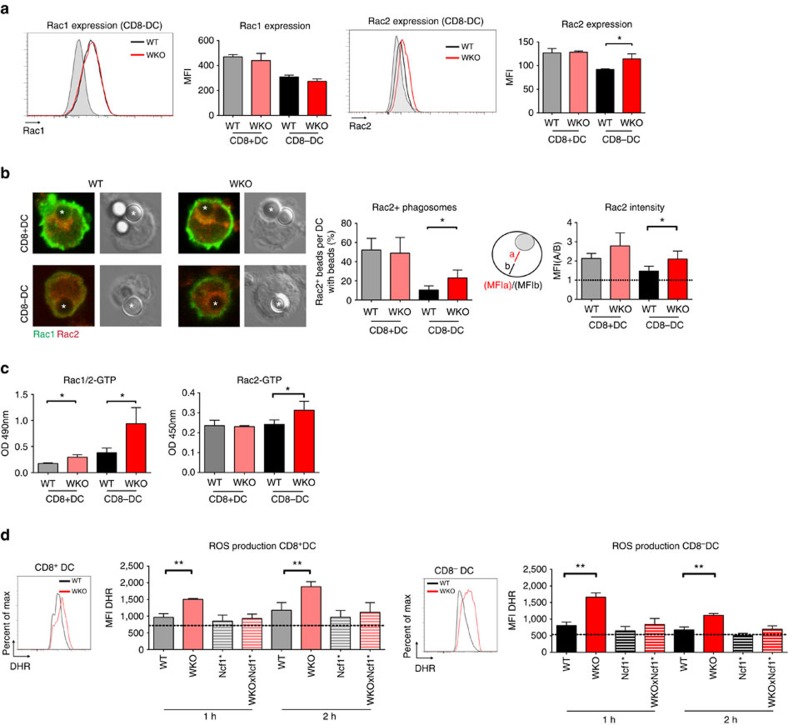
WASp KO CD8^−^ DCs activate Rac2 and ROS production. (**a**) Rac1/2 expression. Wild-type and WASp KO CD8^+^ and CD8^−^ DCs from mice on C57Bl/6 background were stained intracellularly with anti-Rac1 and anti-Rac2 antibodies and analysed by flow cytometry. Bar represents mean±s.d. of WT *n*=3; WKO *n*=3. (**b**) Rac1/2 localization. DCs were incubated with ovalbumin-coated beads for 2 h to allow phagocytosis, transferred to fibronectin-coated glass and stained for Rac1-FITC (green) and Rac2-Alexa555 (red) antibodies and analysed by confocal microscopy. Both panels show Rac1 and Rac2 staining to the left and bright field to the right, with the phagocytosed bead marked with an asterisk. (Left panel) Rac2 co-localization with the phagosome was calculated as: [(beads with Rac2)/(cells with beads)] × 100. Bars represent mean±s.d. of 3–4 mice; 7–16 pictures with total 19–119 cells per mouse. (right panel) The MFI from the middle of the cell towards the bead (**a**) or in the opposite direction (**b**) was measured using the ImageJ software. The (MFI a/MFI b) is shown as Rac2 intensity around the bead. Bars represent mean±s.d. of 3–4 mice; 3–4 pictures with total 11–21 cells per mouse. (**c**) Rac1/2 activity. Quantification of active GTP-bound Rac1/2 and GTP-bound Rac2. Bars represent mean±s.d. of 3–6 mice. (**d**) NADPH induced ROS production. DCs from wild type, WASp KO, Ncf1*, and WASp KO × Ncf1* mice on C57Bl/6 background were enriched and incubated with DHR-coated beads for 1–2 h and analysed by flow cytometry for ROS production. The dotted line indicates background DHR intensity upon DPI treatment. The data in (**a**) are representative of three and (**b**–**d**) of two separate experiments. *P<0.05; **P<0.01 as calculated by the unpaired Student's *t*-test. Scale bar, 5 μm. MFI, mean fluorescence intensity; WT, wild type; WKO, WASp KO.

CD8^+^ DCs assemble a phagosomal complex consisting of Rac2 and the NADPH complex to maintain a neutral pH of the phagosome^21^,^36^. To study Rac2 expression and co-localization to the phagosome, we incubated wild-type and WASp KO CD8^+^ and CD8^−^ DCs with ovalbumin-coated beads and stained for Rac1 and Rac2. Rac1 expression was higher in CD8^+^ DCs than in CD8^−^ DCs and this was detected both in wild-type and WASp KO DCs (Fig. 7a). Wild-type CD8^+^ DCs had high expression of Rac2 that co-localized with the phagocytosed bead together with the NADPH component gp91phox (Fig. 7a,b, upper left image; Supplementary Fig. 9a–c). Wild-type CD8^−^ DCs had less Rac2 expression and did not co-localize Rac2 around the bead (Fig. 7a,b, lower left image; Supplementary Fig. 9a–c)^21^. WASp KO CD8^+^ DCs showed similar expression and co-localization of Rac2 when compared with wild-type CD8^+^ DCs (Fig. 7a,b, upper right image; Supplementary Fig. 9a–c). The phenotype of WASp KO CD8^−^ DCs was strikingly similar to wild-type CD8^+^ DCs with increased expression of Rac2 and increased co-localization of Rac2 and gp91phox to the phagocytosed bead (Fig. 7a,b, lower right image; Supplementary Fig. 9a–c). To quantify Rac1/2 activity, we measured active GTP-bound Rac1/2 in protein lysates of DCs incubated with ovalbumin-coated beads. Wild-type CD8^+^ DCs had lower GTP-Rac1/2 when compared with WASp KO CD8^+^ DCs (Fig. 7c). When compared with wild-type CD8^−^ DCs, WASp KO CD8^−^ DCs had higher quantity of active GTP-bound Rac1/2 (Fig. 7c). We next quantified Rac2 activity specifically and found that WASp KO CD8^−^ DC had the highest quantity of active GTP-bound Rac2 when compared with wild-type CD8^−^ DCs, and wild-type and WASp KO CD8^+^ DCs (Fig. 7c).

Because Rac2 activity is linked to the assembly of the NADPH complex that directly regulates the production of reactive oxygen species (ROS), we measured ROS in CD8^+^ and CD8^−^ DCs. Enriched DCs from the spleen were incubated with beads coated with dihydrorhodamine 123 (DHR) that emits fluorescence in the presence of ROS. CD8^+^ and CD8^−^ DCs from WASp KO mice showed increased ROS production at 1 and 2 h when compared with corresponding DCs in wild-type mice (Fig. 7d). To confirm that the increased DHR signal was due to increased ROS, DCs were pre-treated with diphenyleneiodonium (DPI) that inhibits ROS production followed by incubation with DHR-coated beads. In the presence of DPI, the DHR signal was quenched in all DCs (indicated as dotted line in Fig. 7d). To understand if the observed increase in ROS production was directly caused by the NADPH oxidase activity, WASp KO mice were bred to Ncf1^m1J/m1J^ mice that have reduced NADPH oxidase activity^37^, herein referred to as Ncf1* mice. WASp KO × Ncf1* mice showed abolished ROS production in CD8^+^ and CD8^−^ DCs and reached the DHR intensity of wild-type CD8^+^ and CD8^−^ DCs (Fig. 7d). This suggests that increased ROS production in WASp KO DCs is dependent on a functional NADPH oxidase. Moreover, reduced NADPH activity in WASp KO × Ncf1* CD8^−^ DCs restored the capacity to acidify phagosomes to pH 3–5 as measured by beads coated with pH rodo-ovalbumin (Supplementary Fig. 10). Together, our data shows that WASp KO CD8^−^ DCs adapt to a CD8^+^ DC phenotype with increased expression of Rac2 that co-localizes with the phagosome and induces elevated ROS production.

### WASp and the WASp-VCA domain in cross-presentation

**Figure 8:**
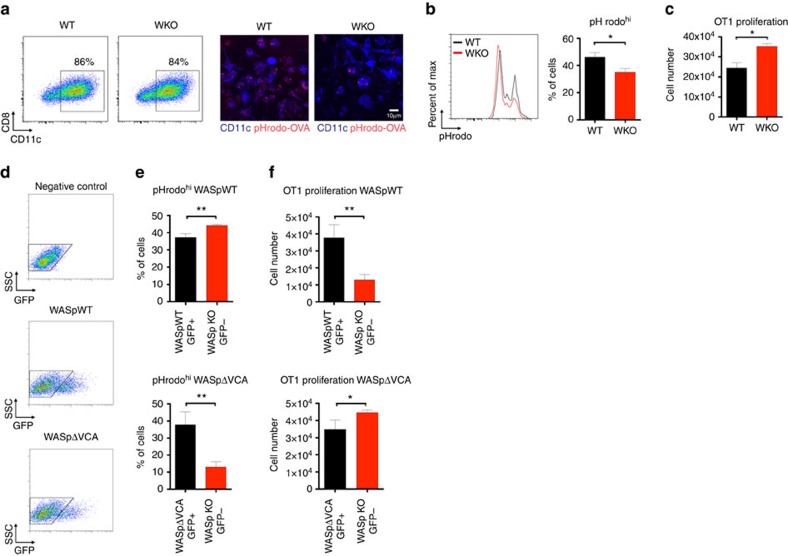
WASp and the WASp-VCA domain in cross-presentation. (**a**–**c**) Wild-type and WASp KO BM DCs from mice on C57Bl/6 background were analysed for capacity to acidify pH rodo-ovalbumin and induce proliferation of OT-I (Vβ5.1/5.2^+^) CD8^+^ T cells. (**d**–**f**) Wild-type BM DCs at day 6 of culture were treated with CK666 to inhibit activity of the Arp2/3 complex or treated with α-amanitin to inhibit polymerase II gene transcription. (**g**,**h**) WASp KO BM DCs at day 6 of culture were Amaxa transfected with wild-type WASp (GFP-WASpWT) or WASp lacking the VCA domain (GFP-WASpΔVCA) and sorted into GFP^+^ and GFP^−^ cells 6 h after transfection. (**a**) Expression of CD11c and CD8 on BM DCs at day 6 of culture and analysis of acidification using uptake of soluble pH rodo-ovalbumin and confocal microscopy. (**b**,**e**,**h**) DCs were incubated with pH rodo-ovalbumin-coated beads and gated for DCs that had taken up one bead and pH rodo intensity was analysed by flow cytometry. (**c**,**f**,**i**) DCs were incubated with 0.5 mg ml^−1^ soluble ovalbumin and co-cultured with OT-I (Vβ5.1/5.2^+^) CD8^+^ T cells, and proliferation was assessed as total number of OT-I CD8^+^ T cells by flow cytometry. (**d**) Wild-type BM DCs were treated for 6 h with CK666 to inhibit Arp2/3 activity and α-amanitin to inhibit RNA polymerase II transcription. Note the drop in polymerized actin measured using phalloidin upon CK666 treatment and the reduced expression of cyclin A2 upon α-amanitin treatment. (**g**) Gating strategy for sorting of GFP^+^ and GFP^−^ WASp KO BM DCs. Negative control indicates non-transfected cells. The data in (**a**–**c**) are representative of three and (**d**–**h**) of two separate experiments. *P<0.05; **P<0.01 as calculated by the unpaired Student's *t*-test. Scale bar, 10 μm. WASpΔVCA, WASp lacking the verprolin-cofilin-acidic domain; WT, wild type; WKO, WASp KO.

To address if WASp directly reduces cross-presentation in DCs, we used the fact that bone marrow (BM)-derived DCs almost exclusively consists of CD11c^+^CD8^−^ DCs. BM-derived DCs from wild-type and WASp KO mice showed similar proportion of CD11c^+^CD8^−^ DCs (Fig. 8a). Similar to WASp KO spleen CD8^−^ DCs, WASp KO BM DCs had reduced capacity to acidify antigen and induced increased proliferation of OT-I CD8^+^ T cells when compared with wild-type BM DCs (Fig. 8b,c). To test if re-expression of WASp would reduce cross-presentation, WASp KO BM DCs were transfected with GFP-WASpWT and GFP^+^ and GFP^−^ cells were sorted after 6 h (Fig. 8d). WASp KO BM DCs expressing GFP-WASpWT showed increased acidification and lower induction OT-I CD8^+^ T-cell proliferation when compared with GFP^−^ WASp KO BM DCs (Fig. 8e,f). Finally, we tested if the WASp-VCA domain was required to dampen cross-presentation. WASp KO BM DCs expressing GFP-WASpΔVCA showed increased acidification and lower induction OT-I CD8^+^ T-cell proliferation when compared with GFP^−^ WASp KO BM DCs (Fig. 8e,f). Together, this data suggests that WASp directly reduced cross-presentation in CD8^−^ DCs in a process independent of the WASp-VCA domain.

## Discussion

Studies of WAS patient cells and WASp-deficient mice have provided critical insights into the role of cell trafficking and cell-to-cell communication during an immune response^1^,^2^,^3^. Decreased migratory or cell-to-cell interaction responses in WASp-deficient cells have been interpreted to mean that WASp directly regulates these responses in WASp-sufficient cells. The results presented in the current study provide an alternative explanation. We provide evidence for that WASp deficiency skews intracellular signalling to Rac2 activation that locally maintains a near neutral pH of endosomes and phagosomes, necessary for cross-presentation. We propose that deletion of key proteins, such as WASp, in patients and mice may induce alternative signalling pathways for cell survival and function that leads to altered biological responses.

WASp serves an important role in the immunological synapse between DCs and CD4^+^ T cells. WASp KO CD4^+^ T cells display normal immunological synapse formation but are unable to reform the immunological synapse after each migration phase^38^. At the DC side of the synapse, WASp KO bone marrow-derived DCs form less stable interactions with ovalbumin-specific wild-type CD4^+^ T cells *in vitro* resulting in decreased T-cell activation^39^. The role of WASp in CD8^+^ DCs has been addressed by direct targeting of ovalbumin to the DEC205 receptor, uniquely expressed on CD8^+^ DCs. Using this approach, wild-type CD8^+^ DCs induced long-lasting contacts with ovalbumin-specific wild-type CD8^+^ T cells *in vivo*, while WASp KO CD8^+^ DCs formed much shorter contacts, suggesting decreased activation of CD8^+^ T cells^13^. However, this study did not distinguish between changes in uptake of ovalbumin complexed with anti-DEC205 antibodies by WASp KO CD8^+^ DCs and changes in presentation of MHC class I—ovalbumin peptides by WASp KO CD8^+^ DCs^13^. In the present study we show that both WASp KO CD8^+^ and CD8^−^ DCs had reduced capacity to take up IgG-ovalbumin immune complexes via Fc receptors, whereas uptake of soluble ovalbumin was similar to wild-type DCs. *In vivo*, serial brief contacts (min) between antigen-presenting DCs and CD8^+^ T cells induce early CD8^+^ T-cell activation, proliferation, and differentiation into effector cytotoxic T cells^40^. However, long-lasting contacts (hours) are needed to form CD8^+^ memory cells in response to antigen^40^. WASp KO CD8^+^ DCs form immune synapses with CD8^+^ T cells that are short-lived *in vivo*^13^, and we show in the present study that WASp KO CD8^+^ and CD8^−^ DCs formed immune synapses *in vitro* leading to increased activation and proliferation of CD8^+^ T cells. Upon *L. major* infection, we showed that despite lower number of CD8^+^ DCs with capacity to cross-present antigen in the dLNs, WASp KO mice had increased activation of dLNs CD8^+^ T cells. Moreover, we detected a consistent increase in CD8^+^ T cells over CD4^+^ T cells in secondary lymphoid organs of WASp KO mice irrespective of inflammatory challenge. This finding suggested that the milieu in WASp KO mice favors CD8^+^ T-cell homoeostasis. However, WASp KO CD8^+^ T cells fail to respond efficiently when specific antigens are presented during viral infections as shown by other groups^25^,^26^,^41^. An explanation for these seemingly contradictory findings is that polyclonal activation of CD8^+^ T cells in WASp KO mice impedes the expansion of antigen-specific CD8^+^ T cells during viral infection. On the basis of the previous studies and the findings in the present study, we propose that downregulation of cross-presentation by WASp may be an active process that is essential to prevent over-activation of CD8^+^ T cells.

During contact eczema, activated effector CD8^+^ T cells are recruited into the skin where they initiate the inflammatory cascade by inducing apoptosis of keratinocytes^42^. Allergen-carrying Langerhans cells induce tolerance upon migration to the dLNs where allergen-specific CD8^+^ T cells are deleted and regulatory T cells activated^43^. In the present study, WASp KO mice challenged with Der p 2 had increased accumulation of DCs in the dermis including the CD11c^+^EpCAM^−^Langerin^−^ DCs that can cross-present antigens. Using *L. major* infection, we detected fewer migratory MHC class II^high^ and CD103^+^ DCs in the dLNs in WASp KO mice. Moreover, we found increased expansion of WASp KO CD8^+^ T cells that were prone to produce IFNγ both *in vivo* and *in vitro*. A unifying hypothesis is that skin pathology in WASp deficiency may result from decreased egress of Langerhans cells and dermal DCs from the skin, increased activation of CD8^+^ T cells with high capacity to producing IFNγ in the skin and dLNs, and decreased suppressive function of WASp KO regulatory T cells^5^,^6^,^7^,^8^. Local accumulation of DCs in the dermis, as we show in the present study, with capacity to cross-present antigen and activate CD8^+^ T cells would worsen this vicious cycle of CD8^+^ T-cell activation in WASp KO mice.

Our data from re-expression of WASp in WASp KO BM-derived DCs suggests that WASp directly reduced cross-presentation in CD8^−^ DCs in a process independent of the WASp-VCA domain. The WASp-VCA domain is also dispensable for WASp activity in T-cell receptor-mediated transcriptional activation^44^,^45^. Future studies are needed to address what part of WASp that is important for antigen processing and presentation. The WASp family of proteins have redundant and unique activities within the cell. N-WASp activity can compensate for critical functions of WASp during lymphocyte development since deletion of both WASp and N-WASp in B or T cells leads to severely compromised development and function^46^,^47^. Another example of such compensatory mechanism comes from studies of WASp KO NK cells. Treatment with IL-2 restores normal cytotoxicity of WASp KO NK cells by increased activation of the WASp-family protein WAVE2 (ref. 48). We have now identified another such compensatory mechanism in which WASp KO CD8^−^ DCs adapt to a CD8^+^ DCs phenotype by increasing Rac2 expression and localizing Rac2 to phagosomal membranes. Importantly, CD8^−^ DCs constitutes up to 40% of DCs in the spleen, as compared with 10% CD8^+^ DCs, and in the setting of wild-type T cells in DC/cWKO mice, WASp KO DCs induced marked expansion of CD8^+^ T cells. While WASp-deficient CD4^+^ T cells are inherently hyporesponsive^49^,^50^,^51^,^52^, recent studies raises an emerging view in which WASp deficiency affects specific cells differently. In fact, WASp deficiency in plasmacytoid DCs^53^, B cells^35^,^54^,^55^,^56^ and DC-mediated activation of CD8^+^ T cells (this study) induce hyper-responsive cells that become subjected to cellular exhaustion. Our data has implications for the treatment of WAS patients and raises concerns for those patients that have limited myeloid reconstitution and normal T-cell reconstitution after BM transplantation and gene therapy^57^,^58^,^59^.

## Methods

### Mice

All mice were bred and maintained in the same room at the animal facility at the Department of Microbiology, Tumor and Cell Biology, Karolinska Institutet, under specific pathogen-free conditions. WASp KO mice on C57Bl/6 and Balb/c background were backcrossed for at least nine generations. Balb/c, C57Bl/6, WASp KO Balb/c, WASp KO C57BL/6, DC/cWKO C57BL/6, OT-I Rag1^−/−^ C57BL/6, OT-II C57BL/6, C57BL/6 J-Ncf1^m1J^/^m1J^ (referred as Ncf1* in the text), WASp KO C57BL/6 × C57BL/6 J-Ncf1^m1J^/^m1J^ mice (referred as WASp KO × Ncf1* in the text) were used at 6–13 weeks of age. The DC/cWKO colony was maintained by breeding WASp^fl/fl^CD11c^wt/wt^ females to WASp^fl/y^CD11c^cre/wt^ males or WASp^fl/fl^CD11c^cre/wt^ females to WASp^fl/y^CD11c^wt/wt^ males. C57BL/6 J-Ncf1^m1J^/^m1J^ mice were purchased from Jackson and backcrossed to WASp KO C57BL/6 mice to generate WASp KO × Ncf1* homozygous mice. All wild-type mice were littermate controls from heterozygous breedings with the respective gene-targeted allele and genotyped before use. Animal experiments were performed after approval from the local ethical committee (the north Stockholm district court).

### Der p 2 challenge protocol and *in vivo* migration

Wild-type and WASp KO Balb/c mice were challenged with Der p 2 (ref. 29). Briefly, mice were shaved on the back and patched 3 × 4 days with 50 μg of Der p 2 in 100 μl of PBS on 1 cm^2^ of shaved skin and sacrificed at day 50. We have used two different preparations of Der p 2 with <35 ng of lipopolysaccharide per mg protein and obtained similar results for the *in vivo* and *in vitro* experiments using both batches. Unchallanged mice were not patched or shaved. For epidermal sheets, full-thickness skin from back was incubated for 90 min at 37 °C in a PBS solution plus 2.5 mg ml^−1^ dispase (Invitrogen). Single cell suspensions for dLN and spleen were prepared by incubating SPL and dLNs in 1% serum complete medium plus 0.5 mg ml^−1^ collagenase D for 30–45 min or plus 1.5 mg ml^−1^ collagenase D for 1.5 h. Ears were further processed using a BD Medimachine system (BD Biosciences) for tissue grinding. Single cell suspensions from spleen were cultured in complete RPMI medium with Der p 2 or PMA and ionomicin (Sigma), and incubated at 37 °C for 4–48 h. Golgiplug (BD Biosciences) was added to the last 4 h of incubation to all cell cultures. For *in vivo* migration, Aldara 5% cream (imiquimod; Meda) was applied epicutaneously on ears of anesthetized mice and let dry to induce dendritic cell maturation and migration to the dLNs. Draining LNs were treated with collagenase D as described above for single cell suspension and analyzed by FACS.

### *L. major* infection

*L. major* strain Fv1 (MHOM/IL/80/FN) was grown at 25 °C to stationary phase in complete M199 supplemented with 20% foetal calf serum (parasite growth medium). Parasites were enriched for infectious metacyclic promastigotes by Ficoll 400 gradient separation^33^. Estimation of parasite number was determined by serial 1:2 dilutions of tissue homogenates in parasite growth medium. The number of viable parasites in each sample was calculated based on the highest dilution at which promastigotes could be grown out after 4–5 days of incubation at 25 °C. The limitation of the assay was 10 parasites per ear and 20 parasites per dLN. Wild-type and WASp KO Balb/c mice were infected intradermally in both ears with 4 × 10^4^ metacyclic *L. major* promastigotes in 10 μl DMEM. Control mice received sham injections of DMEM. Progression of lesion development was measured weekly using a digital veiner calliper and indicated as diameter of the lesion. After 2 or 6 weeks mice were euthanized, and ears and retromaxillar dLNs were removed. Single cell suspensions were prepared by incubating ears and dLNs in 1% serum complete medium plus 1.5 mg ml^−1^ collagenase D for 1.5 h or plus 0.5 mg ml^−1^ collagenase D for 30–45 min, respectively. Ears were further processed using a BD Medimachine system (BD Biosciences) for tissue grinding. To measure cytokine production, single cell suspensions from retromaxillar dLNs were cultured with PMA and ionomicin (Sigma) and golgiplug (Becton Dickenson) for 4 h.

### Antibodies

The following antibodies were used for flow cytometry and/or immunohistochemistry: CD3-PE/Cy7 1:100 (557851), CD8α-PerCP/Cy5.5 1:100 (100734), CD8α-Alexa647 1:200 (557682), CD11c-biotin 1:200 (553800), CD45-APC/Cy7 1:200 (557659), CD45-PerCP/Cy5.5 1:200 (103236), CD62L-FITC 1:100 (553150), IgG1-FITC 1:100 (553443), IgE-biotin 1:200 (553419), PDL1-PE 1:200 (558091), B220-V500 1:200 (561227), CD3-V500 1:200 (560773), Vα2-FITC 1:100 (553288), Vβ5.1/5.2-PE 1:200 (553190), CD86-FITC 1:200 (553691), CD69-PE 1:200 (553237; BD Biosciences), CD4-eFluor450 1:100 (48-0042-82), CD4-APC 1:200 (100516), CD8β-biotin 1:200 (13-0083-81), CD103-FITC 1:200 (121420), F4/80-eFluor450 1:100 (48-4801-82), CD11c-PE/Cy7 1:100 (117318), MHCI-eFluor450 (H2-Kb) 1:100 (48-5958-80), IFNγ-PerCP/Cy5.5 1:100 (505822), IL-17A-FITC 1:100 (11-7177-81; eBioscience), Strepavidin-alexa555 1:1000 (S32355) (ThermoFisher), 7AAD 7 μl per 200 μl of cell volume (51-2359KC) (BD Pharmingen), Live/Dead Fixable Vivid-nearIR 1:500 (L10119), live/dead-AmCyan 1:400 (L34966) (Invitrogen), B220-APC 1:400 (103212), B220-Pacific Blue 1:200 (103227), EpCAM-APC 1:200 (118214), CD11b-PerCP/Cy5.5 1:100 (101228), CD4-FITC 1:100 (100510), MHCII-APC (I-A/I-E) 1:400 (107614), TCRβ-APC 1:200 (109212), B220-APC/Cy7 1:200 (103224), FcγRI 1:100 (139303), FcγRII/III 1:100 (101308), gp91phox 1:200 (650102), CD44-PE 1:400 (103008) (BioLegend), CD19-APC 1:200 (LS-C148489) (LSBio), CD207(langerin)-FITC 1:400 (DDX0362A488-100) (Dendritics), rac1-FITC 1:50 (bs-4186R-FITC), rac2-Alexa555 1:50 (bs-6153R-A555) (BIOSS), biotin-XX phalloidin 1:1000 (B7474) (Invitrogen), γ-tubulin 1:200 (072M4808) (Sigma), FcRn 1:100 (AF6775) (R&D Systems), and cyclin A2 1:200 (ab38) (Abcam), aWASp 1:200 (sc-13139), aGAPDH 1:200 (sc-25778) (Santa Cruz).

### Histology and flow cytometry

For Der p 2-challenged mice, the skin within the 1 cm^2^ square shaved and treated with Der p2 was macroscopically examined, and 4 mm^2^ punch biopsies were taken from areas of red thickened skin for sections and image analysis. Skin tissues were snap frozen in Tissue-Tek OCT (Bio-Optica). Sections (8–10 μm) were cut, air dried overnight and fixed in cold acetone. Hematoxylin and eosin staining was performed using standard protocol and immunohistochemistry^46^. Images were acquired using a Leica DM IRBE confocal laser scanning microscope (Leica Microsystems) equipped with 1 argon and 2 HeNe lasers, using an HC PL APO lens at 20 × /0.70 CS and 63 × /1.32 IMM CORR oil and 90% glycerol (MP Biomedicals). Images were processed with Adobe Photoshop CS4 Version 11.0.2 (Adobe Systems) and ImageJ software. Histological examination and analysis of skin sections were performed blindly where the identity of the section was unknown to the observer. The epidermis thickness values represent the picture with the highest measured thickness of each mouse. When counting cells on images, co-localization of two fluorochromes was based on colour intensity between 150–255 pixel co-localization of red, green, and/or blue using the ImageJ software. To quantify the cells per area on epidermis or in the dermis of whole-skin sections, the cells were counted and areas measured using ImageJ. For flow cytometry analysis, single cell suspensions from organs were prepared and erythrocytes lysed with ACK buffer. Cells were immunolabelled and acquired using a FACS Aria or LSR Fortessa (Becton Dickenson). Analyses were made using FlowJo software (version 7.2.5 TreeStar Inc.).

### Immune synapse and cross-presentation assay

For immune synapse experiments, the splenic DC population was expanded by subcutaneous injection of 1 × 10^6^ Flt3-ligand B16 melanoma cells and DCs purified after 7–10 days with the CD8^+^ DC isolation kit followed by CD11c positive selection (Miltenyi Biotec). CD8^+^ and CD8^−^ DCs were pulsed with ovalbumin overnight and incubated with OT-I CD8^+^ T cells for 2 h. Cells were transferred to fibronectin-coated slides, fixed and stained with γ -ubulin (green) and actin (red). For cross-presentation assays, CD8^+^ DCs and CD8^−^ DCs from wild-type and WASp KO C57Bl/6 mice were isolated by incubating spleens with complete medium plus 1% serum and 0.5 mg ml^−1^ collagenase D followed by enrichment with the Dynabeads mouse DC enrichment kit (Invitrogen) and thereafter FACS sorted based on CD8 and CD11c using FACS Vantage. FACS-sorted DCs were pulsed with 16, 50 and 150 μg ml^−1^ soluble ovalbumin, with 3 μm ovalbumin-coated latex beads (Life Technologies), or with 2 μg ml^−1^ SIINFEKL peptide overnight. To examine cross-presentation of immune complexes (ICs), sorted DCs were incubated with pre-formed ICs of anti-TNP-IgG1 and TNP(5)-OVA at 0.25, 1.25 and 6.25 μg ml^−1^ final concentrations. OT-I CD8^+^ T cells from spleen were sorted by negative selection using the CD8α T-cell isolation kit II (Miltenyi Biotec), with a purity of more than 95%, and labelled with 2 μM CFSE (Invitrogen). Sorted DCs were co-cultured with OT-I CD8^+^ T cells at 1:10 DC:T-cell ratio and analyzed by FACS after 72 h. For the ^3^H-thymidine incorporation assay, OT-I T cells were co-cultured with CD8^+^ or CD8^−^ DCs for 72 h and pulsed with 1 μCi of ^3^H-thymidine for the last 12 h, collected and scintillation measured.

### Antigen processing and ROS production assay

The splenic DC population was expanded by subcutaneous injection of 1 × 10^6^ Flt3-ligand B16 melanoma cells and DCs purified after 7–10 days with the CD8^+^ DC isolation kit followed by CD11c positive selection (Miltenyi Biotec). Enriched CD8^+^ DCs and CD8^−^ DCs were incubated with 16, 50 and 150 μg ml^−1^ ovalbumin-Alexa594 or DQ-ovalbumin (Invitrogen) for up to 6 h for ovalbumin uptake and degradation, respectively. To assess acidification capacity, ovalbumin was linked to pH-rodo according to the manufacturer's instructions (Invitrogen) and DCs were incubated with 50 μg ml^−1^ ovalbumin-pH rodo for 6 h. To measure the production of ROS, the probe DHR (Life Technologies) was used. To control the amount of ovalbumin-pH rodo or DHR taken up over time by each cell, a particulate antigen assay was performed by coating 3 μm latex beads (Life Technologies) to pH rodo-ovalbumin or DHR. DCs that took up only one bead were gated on a flow cytometer according to FSC versus SSC parameters for acidification analysis. As negative controls for acidification and for ROS production, DCs were either pre-treated with 20 mM of NH_4_Cl before addition of the pH rodo-ovalbumin-coated beads, or with 20 μM DPI before addition of the DHR-coated beads. Upon incubation, cells were stained with CD11c, MHCII and CD8 to identify CD11c^+^MHCII^+^CD8^+^ DC and CD11c^+^MHCII^+^CD8^−^ DCs and other cells including plasmacytoid DCs, B and T cells were excluded in a dump gate using B220 and CD3. Dead cells were excluded based on positive staining for DAPI. The pH was measured with a slightly modified protocol described in Savina *et al*.^36^ In short, DCs were enriched using dynabeads mouse DC enrichment (Life Technologies) and incubated with NHS-ester polyamino beads coupled with 1 mg ml^−1^ FITC and 1 mg ml^−1^ Alexa647. A standard curve was performed where enriched DCs were allowed to phagocytose the beads for 30 min. Cells were fixed with 4% paraformaldehyde, and incubated with 0.1% Triton X-100 in PBS with pH kept at 7. Afterwards DCs were submitted to PBS solutions with pH ranging from 5 to 8 and let incubate for a couple of minutes. Samples were immediately analysed by FACS and MFI of FITC and Alexa647 measured on CD11c^+^-gated cells. The standard curve was obtained by calculating the ratio of ‘MFI FITC/MFI Alexa647' at the corresponding pH. WT and WASp KO DCs were incubated with the same beads used for the standard curve, the MFI for FITC and Alexa647 was obtained on gated CD8^+^CD11c^+^ or CD8^−^CD11c^+^ cells (dump gate used with B220, CD3 and live/dead antibodies) and the pH calculated using the formula obtained by the standard curve. To assess uptake of immunocomplexes (ICs) DCs were enriched as above described and incubated with a 1:20 dilution of pre-formed ICs of anti-TNP-IgG1 and TNP(5)-OVA at 0.25, 1.25 and 6.25 μg ml^−1^ final concentrations. Antigen presentation of DCs in the presence of ICs was determined by flow cytometry and staining of DCs with the antibody towards H-2K^b^-SIINFEKL (clone 25-D1.16, Biolegend).

### Rac1/2 assays

The splenic DC population was expanded by subcutaneous injection of 1 × 10^6^ Flt3-ligand B16 melanoma cells and DCs enriched after 7–10 days with the CD8^+^ DC isolation kit followed by CD11c positive selection (Miltenyi Biotec). To determine Rac1/2 and gp91phox expression and co-localization to the phagosome, enriched CD8^+^ DCs and CD8^−^ DCs were incubated with 3 μm ovalbumin-coated latex beads for 2 h at 37 °C, transferred to glass coverslips coated with 50 μg ml^−1^ fibronectin (Gibco) and incubated for 1 h at 37 °C. Afterwards, cells were fixed with 4% paraformaldehyde, quenched with 0.1 M glycine, permeabilized and stained intracellularly with primary antibodies for Rac1, Rac2 and gp91phox. Cells were analysed using a Zeiss LSM 780 confocal microscope equipped with a diode laser with wavelength 405 nm, an Argon laser with wavelengths 458/488/514 nm DPSS 561 nm and a HeNe 633 nm laser. Pictures were acquired using a Plan-Apo 63 × /1.4 oil-immersion lens and the ZEN Black 2011 software. Rac2 co-localization with the phagosome was calculated as: [(beads with Rac2)/(cells with beads)] × 100. Five to 15 images per mouse were analyzed. Rac2 co-localization was further assessed by z-stack analysis from pictures taken with a 63 × objective and a step-size of 1.3 μm per picture. Pictures were compressed into one single image using ImageJ. For Rac1/2 activity, protein lysates were prepared from DCs incubated with ovalbumin-coated latex beads for 2 h and protein content measured and diluted to equal concentration. Active GTP-bound Rac1/2 was quantified using the G-LISA Rac1,2,3 Activation Assay Biochem Kit (Cytoskeleton). To quantify GTP-bound Rac2 a modified version of the G-LISA kit was used with an anti-Rac2 (Santa Cruz Biotechnology) detection antibody, anti-rabbit-HRP (Santa Cruz Biotechnology) secondary, and anti-goat-HRP (Santa Cruz Biotechnology) tertiary antibodies.

### Transfection of BM DCs

BM cells from femur and tibia were cultured in 20 ng ml^−1^ GMCSF (Peprotech) for 6 days. For confocal microscopy, BM DCs were incubated with 0.5 mg ml^−1^ soluble ovalbumin-pH rodo for 1 h and analyzed by confocal microscopy after staining with anti-CD11c. For re-expression of WASp, WASp KO BM DCs were transfected with eGFP-WASpWT or eGFP-WASpΔVCA constructs^60^ using Amaxa transfection (Primary cell 4D nucleofector kit, Lonza). After 6 h, GFP^+^ and GFP^−^ cells were FACS sorted using FACS Jazz. To assess acidification capacity, BM DCs after Amaxa transfection were incubated with ovalbumin-pH rodo beads for 30 min and pH rodo fluorescence determined in cells that had taken up one bead. To determine proliferation of OT-I CD8^+^ T cells, BM DCs after Amaxa transfection were incubated with 0.5 mg ml^−1^ ovalbumin and LPS overnight. BM DCs were co-cultured with OT-I CD8^+^ T cells at 1:10 DC:T-cell ratio and analyzed by FACS after 48 h.

### Data and statistical analysis

For comparison between wild-type and WASp KO mice, data passed the normality test and was analyzed by the unpaired Student's *t*-test using GraphPad Prism 5.0 software and a two-tailed *P* value with 95% confidence interval was acquired. Data is shown as mean±s.d. and *P*<0.05 was considered significant.

### Data availability

The data that support the findings of this study are available from the corresponding authors on request.

## Additional information

**How to cite this article**: Baptista, M. A. P. *et al*. Deletion of Wiskott–Aldrich syndrome protein triggers Rac2 activity and increased cross-presentation by dendritic cells. *Nat. Commun.* 7:12175 doi: 10.1038/ncomms12175 (2016).

## Supplementary Material

Supplementary Information Supplementary Figures 1-10
