# Supplementary material for: Deletion of Wiskott–Aldrich syndrome protein triggers Rac2 activity and increased cross-presentation by dendritic cells
Source: Nat Commun. 2016 Jul 18;7:12175. doi: 10.1038/ncomms12175 (PMC4960314; doi:10.1038/ncomms12175)
Supplement: Supplementary Information — Supplementary Figures 1-10 [file ncomms12175-s1.pdf]

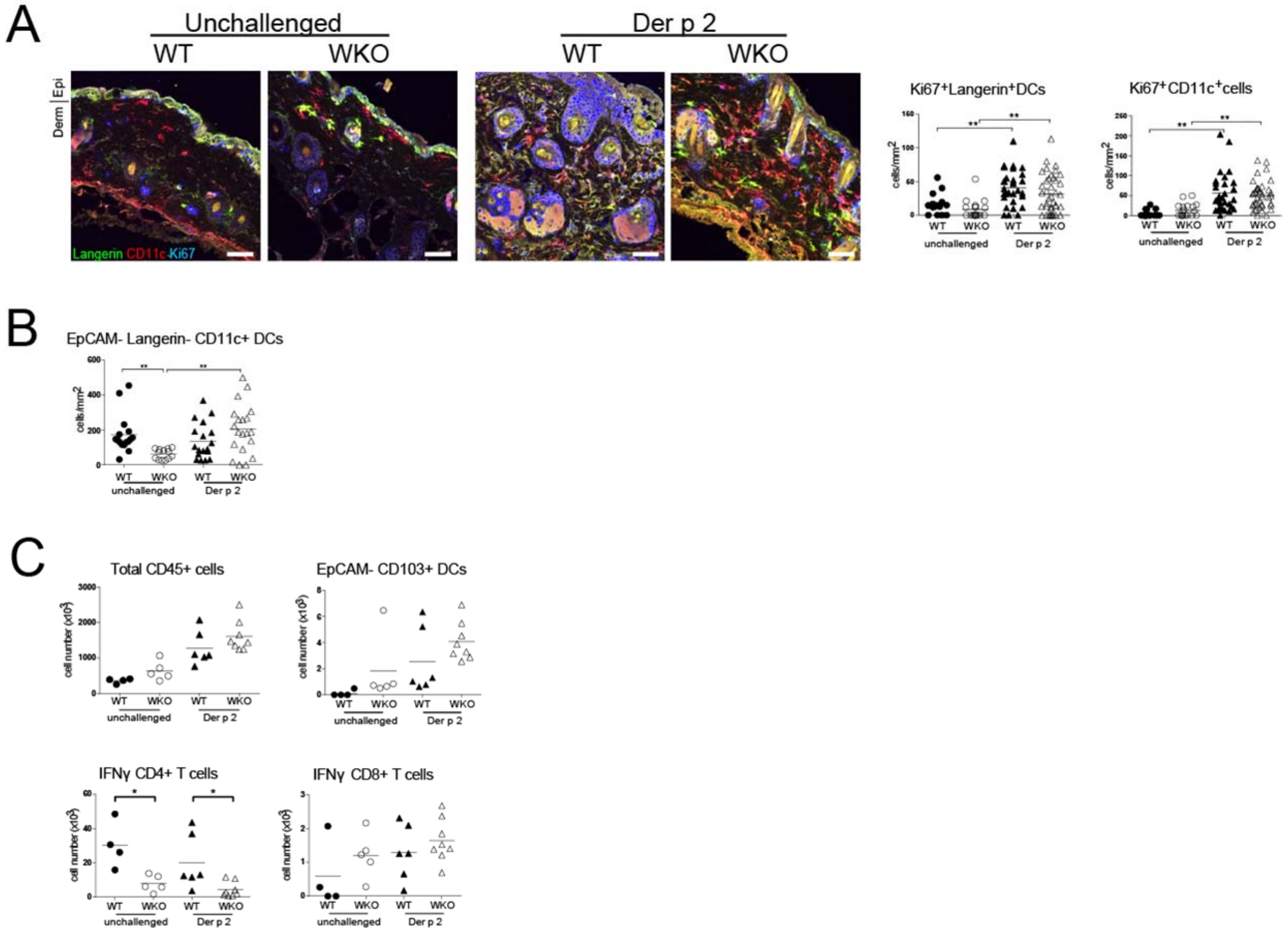

Supplementary Fig. 1. Skin DCs and T cells in WASp KO mice. (**a**) Whole skin biopsies from unchallenged and Der p 2 challenged mice at day 50 were immunolabeled with Langerin (green), CD11c (red) and the proliferation marker Ki67 (blue) to determine the number of proliferating Langerin$^+$Ki67$^+$ and CD11c$^+$Ki67$^+$ DCs. Bar represents mean and each dot one picture. WT unchallenged n=3; WKO unchallenged n=4; WT Der p 2 n=7; WASP KO Der p 2 n=8. (**b**) The mean number of total EpCAM$^-$ Langerin$^-$ CD11c$^+$ DCs, with cross-presenting capacity, per mm^2 dermis is indicated. Each dot represents one figure. (**c**) Absolute numbers of cells in the back skin at day 50 from unchallenged and Der p 2 challenged WT and WASp KO mice as measured by flow cytometry. WT unchallenged n=4; WKO unchallenged n=5; WT Der p 2 n=6; WASP KO Der p 2 n=8. *P<0.05; **P<0.01 as calculated by unpaired Student's t-test. Scale bar: 50 µm. Abbreviations: WT; wildtype, WKO; WASp KO.

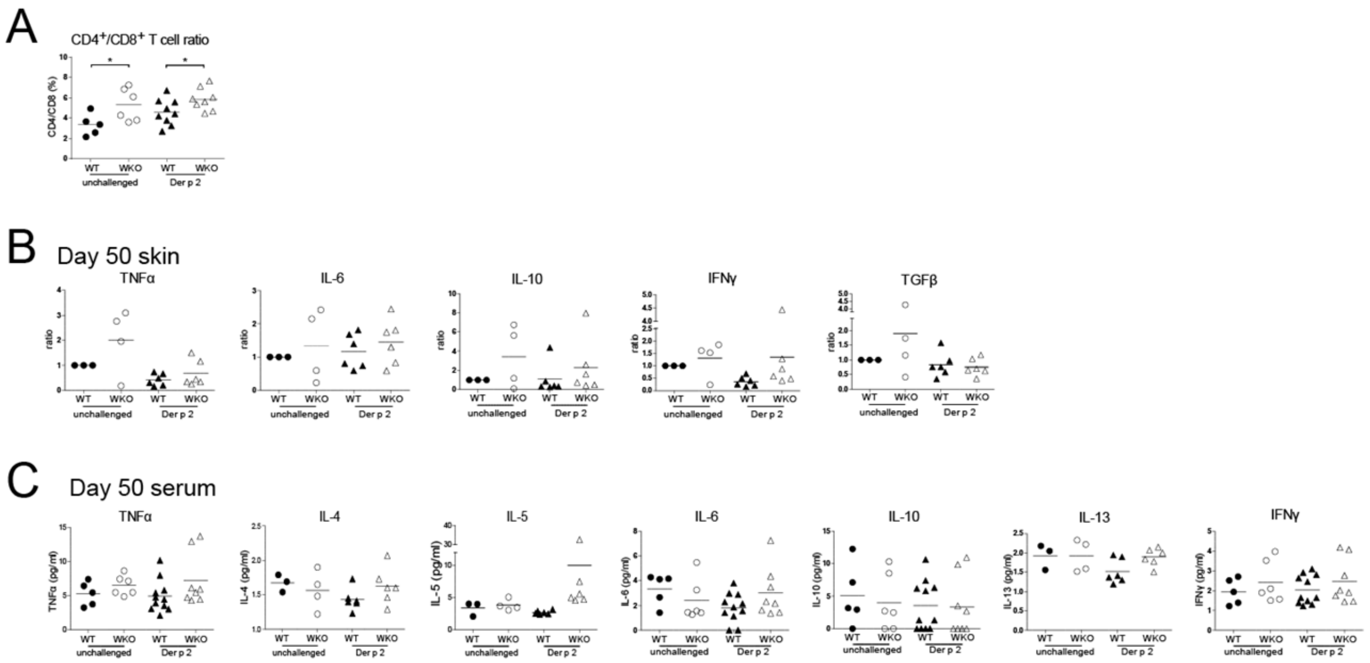

Supplementary Fig. 2: $CD4^{+}/CD8^{+}$ T cell ratio and cytokine profile. (**a**) CD4/CD8 ratio was calculated from flow cytometry analysis of percentage $CD4^{+}$ and $CD8^{+}$ T cells in peripheral blood of unchallenged and Der p 2 challenged WT and WASp KO mice at day 50. Bar represents mean value and each dot represents one mouse. Results represent a pool of two experiments. WT unchallenged n=5; WKO unchallenged n=6; WT Der p 2 n=9; WKO Der p 2 n=8. (**b**) Skin cytokines. TNFα, IL-6, IL-10, IFNγ, and TGFβ from day 50 skin biopsies were measured by real-time qtPCR. (**c**) Serum cytokines. TNFα, IL-4, IL-5, IL-6, IL-10, IL-13 and IFNγ were measured by cytometric bead array in serum from mice at day 50. Bar represents mean and each dot one mouse. Abbreviations: WT; wildtype, WKO; WASp KO.

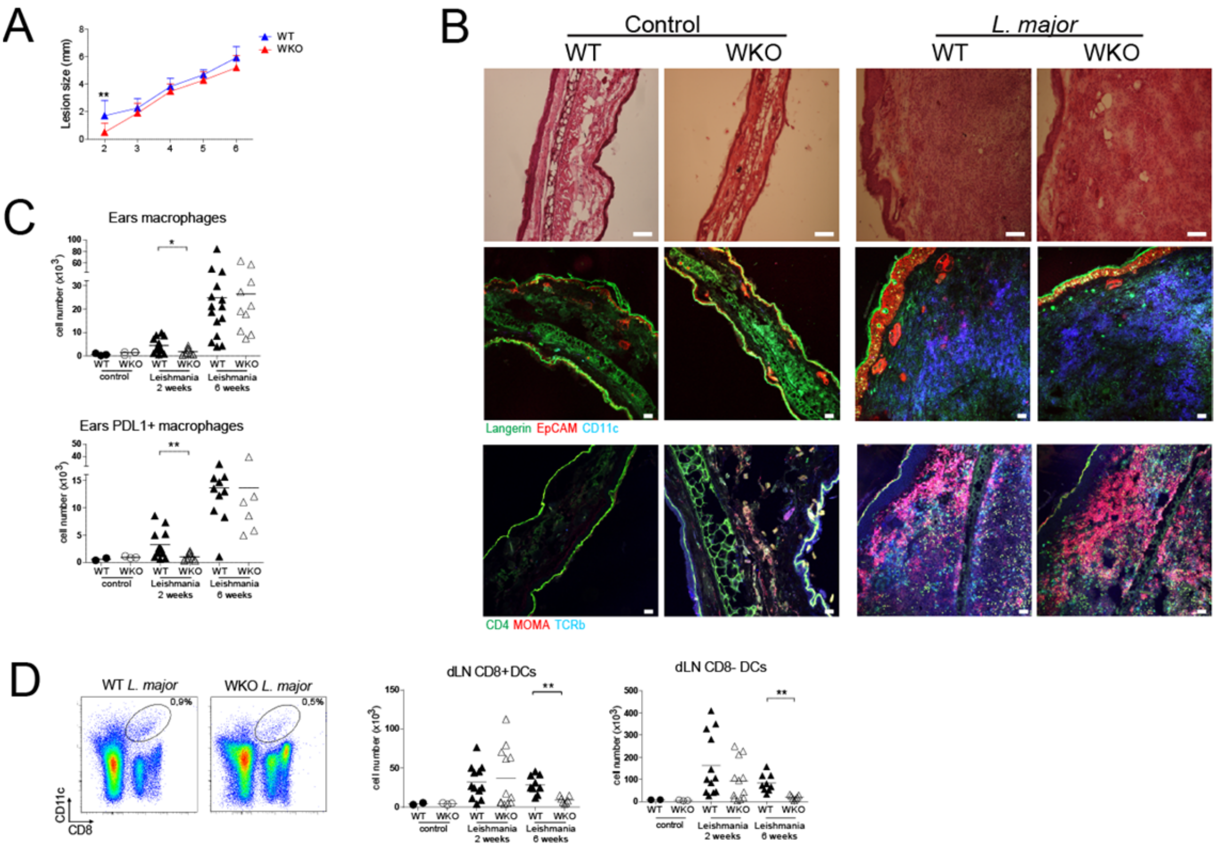

Supplementary Fig. 3. WASp KO mice and *L. major* infection. WT and WASp KO mice were injected with DMEM (control) or *L. major* intradermally in each ear. (**a**) The diameter of the lesion measured weekly is indicated as mean±SD. Blue triangles: *L. major* WT mice; Red triangles: *L. major* WKO mice (**b**) Skin histopathology induced by *L. major*. Ear sections (8 µm) from control and *L. major* infected WT and WASp KO mice from week 6 were stained with: (upper panel) hematoxylin and eosin; (middle panel) CD207 (Langerin, green), CD11c (blue) and EpCAM (red) to detect Langerhans cells (Langerin$^{\pm}$CD11c^{+}EpCAM^{+}) or dermal DCs (Langerin$^{\pm}$CD11c^{+}EpCAM^{-}); and (lower panel) CD4 (green), MOMA (red) and TCRβ (blue) to analyse frequency of CD4^{+} T cells (CD4^{+} TCRβ^{+} cells) and macrophages (MOMA^{+} cells). (**c**) Absolute numbers of cell infiltrates in the ear, including total macrophages (F4/80^{+} cells) and activated macrophages (F4/80^{+}PDL1^{+} cells) at week 2 and week 6 post infection as measured by flow cytometry. (**d**) Mice were sacrificed after 2 and 6 weeks post infection and dLNs were removed for determination of frequency of CD8^{+}CD11c^{hi} and CD8^{-}CD11c^{+} DCs by flow cytometry. (**c-d**) Bar represents mean value and each dot represents one ear or dLN. WT control n=3; WKO control n=3; WT *L. major* 2 weeks n=6; WASp KO *L. major* 2 weeks n=6; WT *L. major* 6 weeks n=7; WASp KO *L. major* 6 weeks n=6-10. *P<0.05; **P<0.01 as calculated by unpaired Student's t-test. Scale bar: 50 µm. Abbreviations: WT; wildtype, WKO; WASp KO.

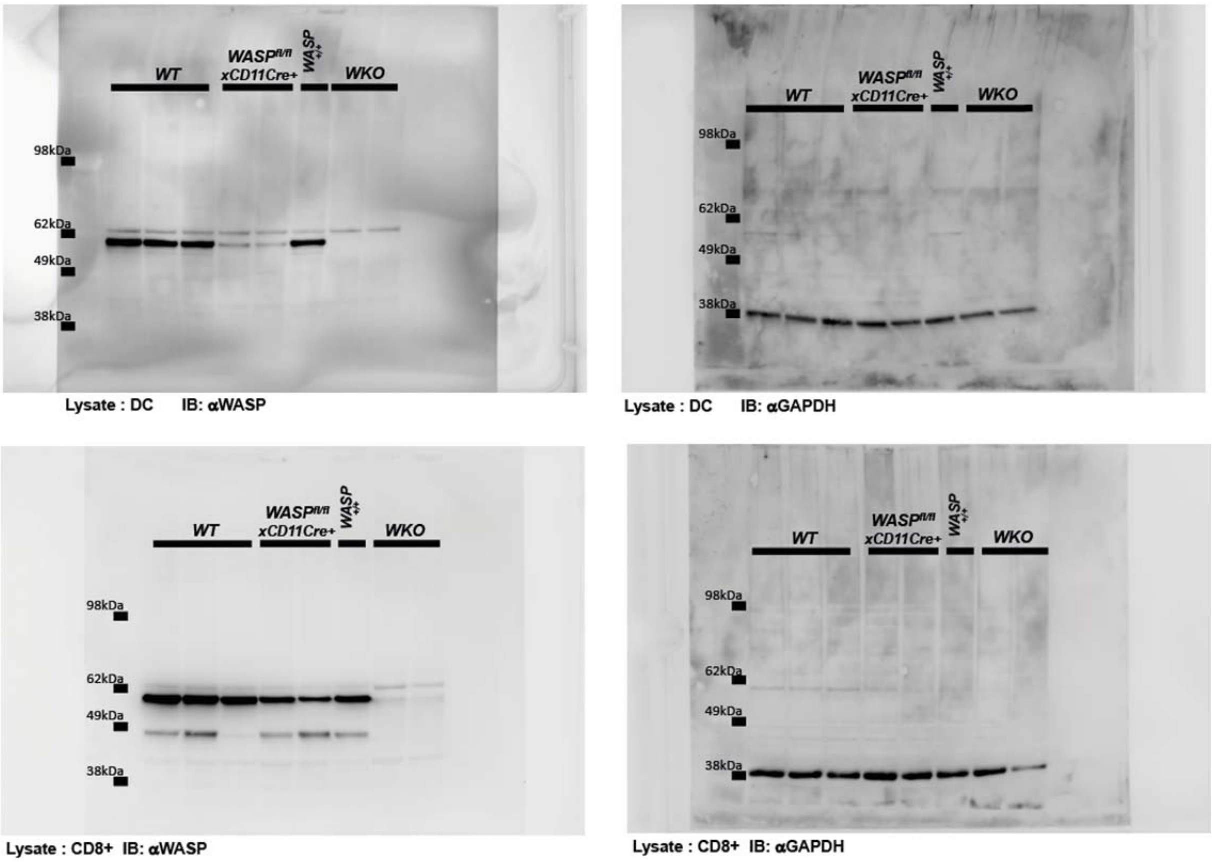

Supplementary Fig. 4. Full-size scans of Western blots shown in Figure 4b. Upper panel shows lysates from spleen DCs. Lower panel shows lysates from CD8^{+} T cells.

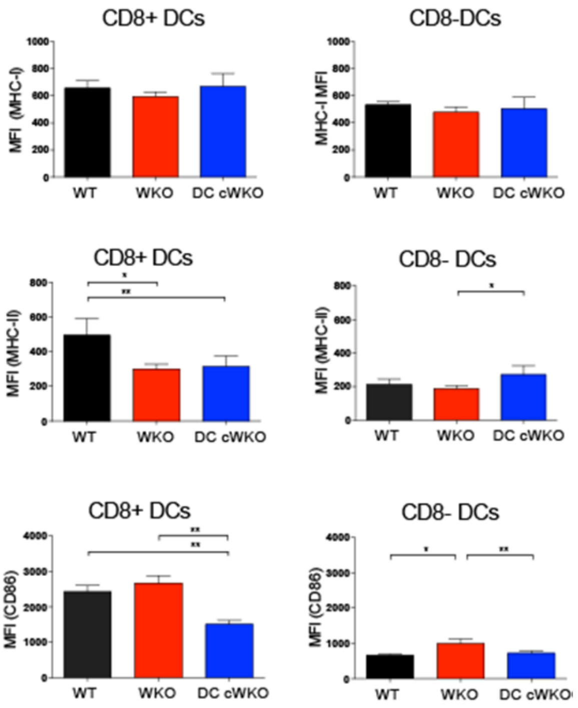

Supplementary Fig. 5. MHC-I, MHC-II and CD86 expression on DCs. Spleen DCs were analysed by flow cytometry to determine expression levels of MHC-I molecules (upper panel), MHC-II (middle panel) and CD86 (lower panel) for $CD8^+$ and $CD8^-$ DCs. Bar represents mean±SD of WT n=3; WKO n=3 and DCcWKO n=6 per group. The data are representative of two similar experiments. Abbreviations: WT; wildtype, WKO; WASp KO; DC/cWKO; $WASp^{fl/fl}CD11c^{Cre/wt}$, fl; floxed (LoxP flanked).

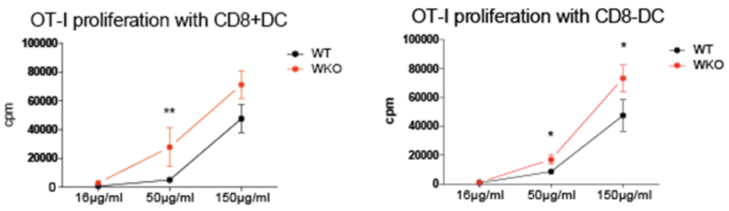

Supplementary Fig. 6. OT-I proliferation estimated by ^{3}H-thymidine incorporation. $CD8^{+}$ and $CD8^{-}$ DCs were co-cultured with OT-I transgenic $CD8^{+}$ T cells for 72 hours and pulsed with ^{3}H-thymidine for the last 12 hours and presented as cpm values. Symbol represents mean±SD of WT n=3; WKO n=3 per group. The data is representative of two separate experiments. *P<0.05; **P<0.01 as calculated by unpaired Student's t-test. Abbreviations: WT; wildtype, WKO; WASp KO.

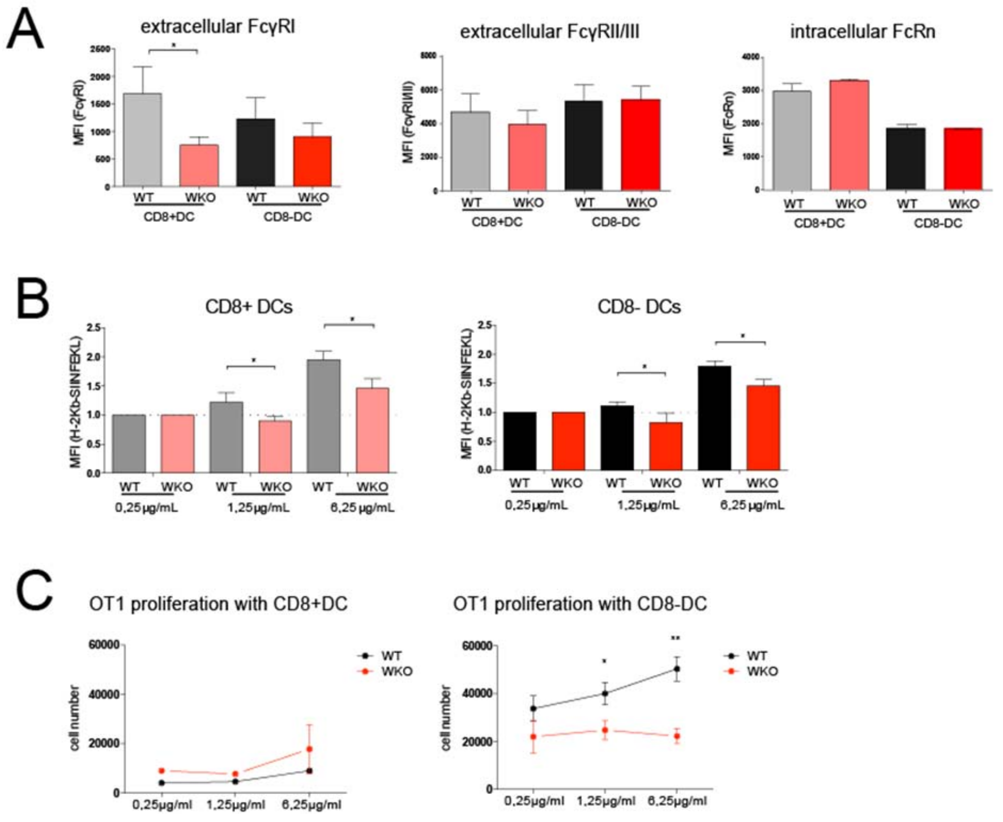

Supplementary Fig. 7. WASp KO DC cross-presentation of immune complexes. (**a**) Fc receptor expression. $CD8^+$ and $CD8^-$ DCs from spleen of WT and WASp KO mice were analysed by flow cytometry for surface expression of FcγRI and FcγRII/II and intracellularly for FcRn. (**b**) MHC I presentation. $CD8^+$ and $CD8^-$ DCs were incubated overnight with pre-formed anti-TNP IgG1 plus TNP(5)-OVA immune complexes containing 0.25, 1.25 or 6.26 µg/ml OVA. Presentation on the surface of SIINFEKL on MHC-I molecules was determined the next day by flow cytometry with the anti-H2K^b-SIINFEKL antibody. (**c**) OT-I $CD8^+$ T cell proliferation. $CD8^+$ and $CD8^-$ DCs were incubated overnight with pre-formed anti-TNP IgG1 plus TNP(5)-OVA immune complexes containing 0.25, 1.25 or 6.26 µg/ml OVA and co-cultured with CFSE labelled OT-I $CD8^+$ T cells. Total number of OT-I $CD8^+$ T cells is indicated after 72 hours. Bars or symbols represents mean±SD of WT n=3; WKO n=3 per group. The data are representative of two similar experiments. Abbreviations: WT; wildtype, WKO; WASp KO.

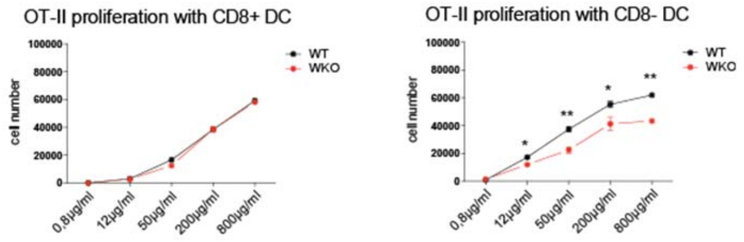

Supplementary Fig. 8. WASp KO DCs and proliferation of OT-II cells. $CD8^+$ and $CD8^-$ DCs were co-cultured with CFSE-labeled OT-II transgenic $CD4^+$ T cells in the presence of different concentration of ovalbumin. OT-II cell number was determined after 72 hours of incubation. Symbol represents mean±SD of WT n=3; WKO n=3 per group. The data is representative of two experiments. *$P<0.05$; **$P<0.01$ as calculated by unpaired Student's t-test. Abbreviations: WT; wildtype, WKO; WASp KO.

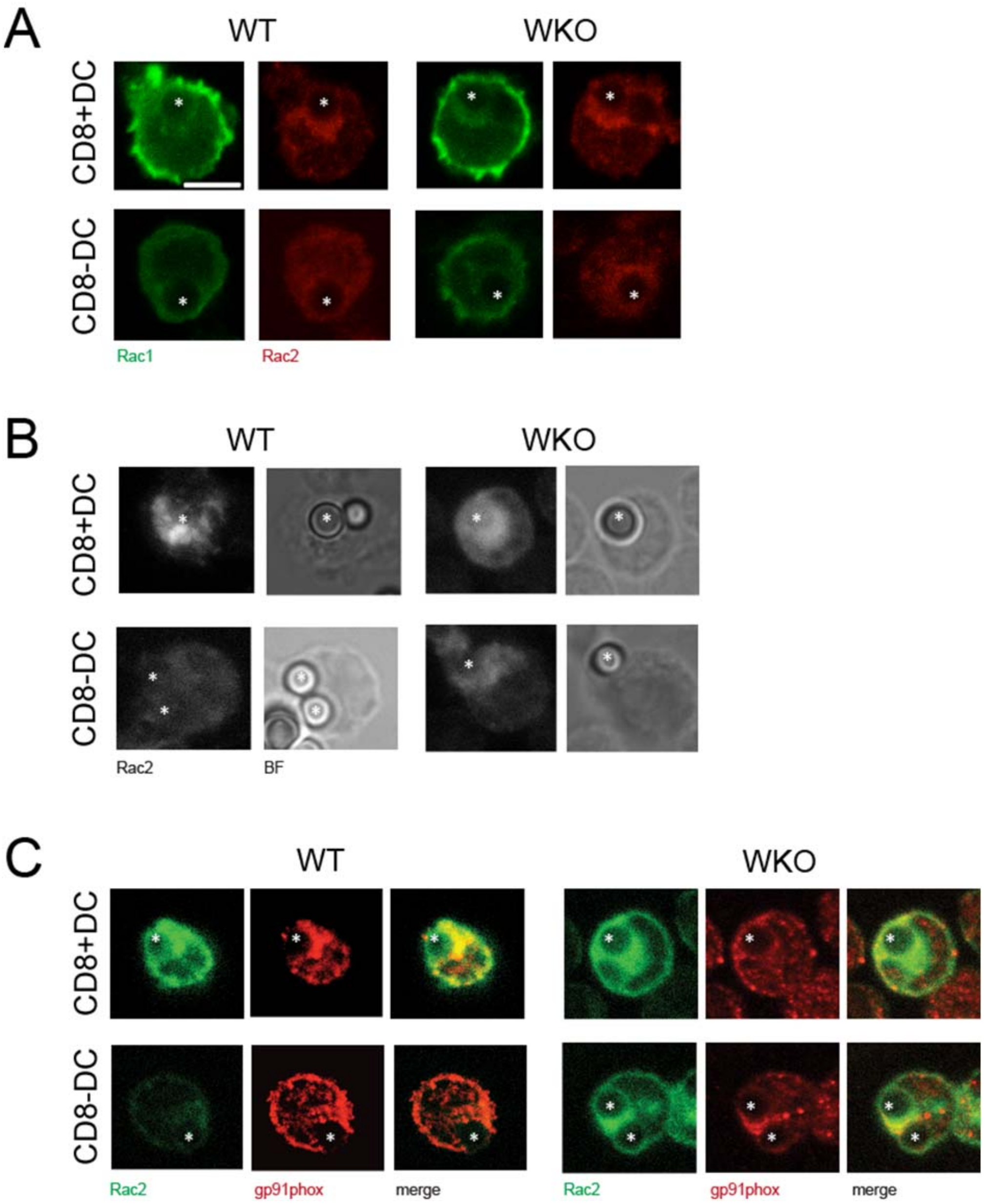

Supplementary Fig. 9. Rac2 and gp91phox co-localization to the phagosome. DCs were allowed to phagocytose ovalbumin-coated latex beads for 1 hour and afterwards to adhere to fibronectin-coated slides. Co-localization to the phagosome of Rac1, Rac2 and gp91phox was analysed by confocal microscopy. (**a**) Rac1 (green) and Rac2 (red) single stainings represented in figure 7A. (**b**) Z-stacks of Rac2 (grey) stainings compressed into one single image from WT and WASp KO DCs. Left panels show Rac2 intensity, right panels show bright field. (**c**) Single focal images of Rac2 (green) and gp91phox (red) single stainings (left and middle panels) and merged (right panel). The asterisk indicates the bead(s) inside the DCs. WT; wildtype, WKO; WASp KO

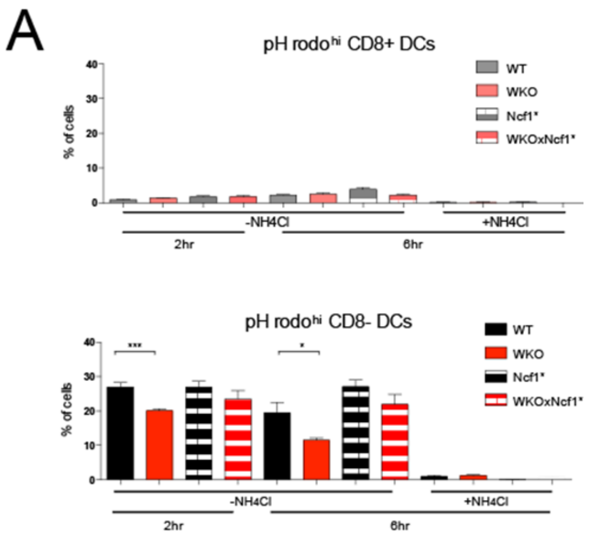

Supplementary Fig. 10. Acidification capacity of the phagosomal compartment by DCs. Wildtype, WASp KO, Ncf1*, and WASpKO x Ncf1* CD8$^+$ and CD8$^-$ DCs from mice on C57Bl/6 background were incubated with pHrodo-ovalbumin-coated beads to assess acidification of antigen in phagocytic vesicles or were pre-treated with NH_4Cl, to abolish acidification before addition of pHrodo-ovalbumin beads. Acidification was assessed by flow cytometry and cells that phagocytosed one bead and showed high intensity of pH-rodo were gated to determine the percentage of cells capable of acidifying the phagosome at the indicated time points. Bar represents mean±SD of WT n=3; WKO n=3. The data are representative of two separate experiments. *P<0.05; **P<0.01 as calculated by unpaired Student's t-test. Abbreviations: WT; wildtype, WKO; WASp KO.
